# Identification and Analysis of NaHCO_3_ Stress Responsive Genes in Wild Soybean (*Glycine soja*) Roots by RNA-seq

**DOI:** 10.3389/fpls.2016.01842

**Published:** 2016-12-09

**Authors:** Jinlong Zhang, Jiaxue Wang, Wei Jiang, Juge Liu, Songnan Yang, Junyi Gai, Yan Li

**Affiliations:** National Key Laboratory of Crop Genetics and Germplasm Enhancement, National Center for Soybean Improvement, Key Laboratory for Biology and Genetic Improvement of Soybean (Ministry of Agriculture), Jiangsu Collaborative Innovation Center for Modern Crop Production, Nanjing Agricultural UniversityNanjing, China

**Keywords:** alkalinity, differentially expressed gene (DEG), Gene Ontology (GO) enrichment analysis, RNA-seq, ion transporter, wild soybean

## Abstract

Soil alkalinity is a major abiotic constraint to crop productivity and quality. Wild soybean (*Glycine soja*) is considered to be more stress-tolerant than cultivated soybean (*G. max*), and has considerable genetic variation for increasing alkalinity tolerance of soybean. In this study, we analyzed the transcriptome profile in the roots of an alkalinity tolerant wild soybean variety N24852 at 12 and 24 h after 90 mM NaHCO_3_ stress by RNA-sequencing. Compared with the controls, a total of 449 differentially expressed genes (DEGs) were identified, including 95 and 140 up-regulated genes, and 108 and 135 down-regulated genes at 12 and 24 h after NaHCO_3_ treatment, respectively. Quantitative RT-PCR analysis of 14 DEGs showed a high consistency with their expression profiles by RNA-sequencing. Gene Ontology (GO) terms related to transcription factors and transporters were significantly enriched in the up-regulated genes at 12 and 24 h after NaHCO_3_ stress, respectively. Nuclear factor Y subunit A transcription factors were enriched at 12 h after NaHCO_3_ stress, and high percentages of basic helix-loop-helix, ethylene-responsive factor, Trihelix, and zinc finger (C2H2, C3H) transcription factors were found at both 12 and 24 h after NaHCO_3_ stress. Genes related to ion transporters such as ABC transporter, aluminum activated malate transporter, glutamate receptor, nitrate transporter/proton dependent oligopeptide family, and S-type anion channel were enriched in up-regulated DEGs at 24 h after NaHCO_3_ treatment, implying their roles in maintaining ion homeostasis in soybean roots under alkalinity. Kyoto Encyclopedia of Genes and Genomes pathway enrichment analysis showed “phenylpropanoid biosynthesis” and “phenylalanine metabolism” pathways might participate in soybean response to alkalinity. This study provides a foundation to further investigate the functions of NaHCO_3_ stress-responsive genes and the molecular basis of soybean tolerance to alkalinity.

## Introduction

Salt-affected soils are major abiotic constraints to crop yield and agricultural sustainability, and can be classified into two main categories: saline and alkaline (Tuyen et al., 2010; Xu and Tuyen do, 2012). Different from salinity, alkalinity is mainly caused by sodium bicarbonate (NaHCO_3_) or sodium carbonate (Na_2_CO_3_), which affects seed germination, plant growth and productivity by the presence of excess Na^+^, combined with HCO_3_^-^, CO_3_^2-^, high pH (>8.5), and poor soil structure. The Food and Agriculture Organization/United Nations Educational, Scientific and Cultural Organization (FAO/UNESCO) showed that alkaline soils cover an area of 434 million hectares (ha) worldwide (FAO, 2000; Munns, 2005). North America and Latin America has 14.5 and 50.9 million ha alkalinity soils, respectively (FAO, 2000; Tuyen et al., 2010). The Song-Nen Plain, a major soybean cultivation region in northeastern China, is one of the top three major contiguous saline-alkali-affected areas in the world, which has an estimated area of 3.73 million ha alkaline soils, and the size of which is increasing by 1.4% annually (Ge et al., 2010; Zhang L.M. et al., 2013). Therefore increasing crop tolerance to alkalinity is essential for food security.

Extensive investigations have been devoted to determine how plants respond to salinity/alkalinity. High concentrations of toxic ions in soils, such as Na^+^ or sometimes Cl^-^, HCO_3_^-^, or CO_3_^2-^, cause an increase in external osmotic pressure and ion imbalance in plants (Serrano and Rodriguez-Navarro, 2001; Zhu, 2001). There is evidence that high soil pH (>8.5) imposes adverse effect on roots, affecting nutrient uptake, disturbing organic acids balance, distribution and accumulation of inorganic ions, especially disrupting cellular pH stability (Chen et al., 2009; Yang et al., 2009; Zhang L.M. et al., 2013; Zhang X. et al., 2013). It has been proposed that there are three major mechanisms for plant salinity tolerance: tolerance to osmotic stress, sodium (Na^+^) exclusion from leaf blades, and tissue tolerance (Munns and Tester, 2008). Osmotic stress inhibits plant growth and causes stomatal closure. An osmotic stress tolerant plant shows less reduction in shoot growth and greater stomatal conductance (Munns and Tester, 2008). Many compatible solutes (such as glycine betaine, proline, sugars, and polyols) are accumulated in plants under salinity, and are essential to balance the osmotic pressure of the cytoplasm (Munns, 2005). Ion exclusion means plants do not accumulate toxic concentration of Na^+^ (Serrano and Rodriguez-Navarro, 2001; Zhu, 2003). Several gene families such as SOS and HKT are involved in ion exclusion mechanisms (Zhu, 2001; Munns, 2005). Tissue tolerance represents a compartmentalization of ions at the cellular level by sequestration of Na^+^ in vacuoles to enhance tolerance to high concentration of ions. Two key genes, *AtNHX1* and *AtAVP1*, are involved in the movement of Na^+^ into vacuoles, affecting tissue tolerance of Arabidopsis (Gaxiola et al., 1999, 2001; Pardo et al., 2006). Under alkaline stress, alkalinity-tolerant plants can uptake nutrients such as iron more efficiently than sensitive plants (Munns, 2005; Peiffer et al., 2012; Xu and Tuyen do, 2012). However, the details about genes and mechanisms of plant tolerance to alkalinity are largely unknown.

RNA-seq has several advantages such as no requirement of prior genome sequence information, higher throughput, wider range of expression levels, and less noise. Therefore it can be used for both model and non-model plant species. RNA-seq has been widely used for transcriptome analysis of crop response to salinity stress, such as wheat (Zhang et al., 2016), rice (Zhou et al., 2016), maize (Zhang L.M. et al., 2013), cotton (Yao et al., 2011), and oilseed rape (Yong et al., 2014). However, fewer studies on RNA-seq analysis of crops response to alkalinity have been reported so far (Fan et al., 2013; Zhang L.M. et al., 2013; DuanMu et al., 2015).

Soybean is one of the most important oilseed crops worldwide, which is rich in vegetable oil, protein and nutraceutical compounds such as isoflavones and saponins (Lam et al., 2010; Schmutz et al., 2010; Korir et al., 2013; Krishnamurthy et al., 2015). It is widely used for human food, animal feed, and industrial products. In ancient China ∼6,000–9,000 years ago, farmers used wild soybean (*Glycine soja*) to select domesticated soybean (Carter et al., 2004; Zhou et al., 2015). Cultivated soybean (*G. max*) has much lower genetic diversity than their wild progenitor (Hyten et al., 2006; Lam et al., 2010; Qi et al., 2014). Wild soybean is generally more tolerant and adapted to biotic and abiotic stress conditions than cultivated soybean. Both species have chromosomes 2*n* = 40 and can be crossed to generate viable, fertile offspring (Xu and Gai, 2003; Ge et al., 2010; Kim et al., 2010; Tuyen et al., 2010). The genomes of both species have been sequenced (Kim et al., 2010; Lam et al., 2010; Schmutz et al., 2010; Li et al., 2014; Zhou et al., 2015), providing useful information for functional genomics studies. However, transcriptome analysis of soybean in response to alkalinity by RNA-seq was limited.

Previous studies on expression profiles of soybean in response to alkaline stress were carried out using hydroponic solution with 50 mM NaHCO_3_ (Ge et al., 2010, 2011), which provides a general knowledge of soybean response to alkalinity. Root is the primary tissue in soil encountering alkaline stress, therefore in this research, we investigated the transcriptome profiles in the roots of an alkalinity-tolerant wild soybean variety, N24852, using quartz sand culture medium subjected to higher concentration of NaHCO_3_ (90 mM), which is more similar to the alkalinity in the field. The aim of this study was to find differentially expressed genes (DEGs), metabolic pathways, and overall transcriptional regulation of soybean response to early stage of NaHCO_3_ stress, which would broaden our understanding of the molecular and regulatory mechanisms of plant response to alkaline stress, and to identify candidate genes that could be utilized to improve soybean tolerance to alkalinity in future breeding programs.

## Materials and Methods

### Plant Growth and NaHCO_3_ Stress Treatment

We identified an alkalinity-tolerant wild soybean variety N24852 from a preliminary screening of 129 soybean varieties (our unpublished data), which showed more alkalinity-tolerant than 95% of the 129 varieties. N24852 is tolerant to biotic and abiotic stresses (Wang et al., 2013) and its alkalinity tolerance was confirmed in this study (Supplementary Figure S1). A widely used salinity-tolerant (but moderately sensitive to alkalinity) cultivated soybean variety Lee 68 (Abel and Mackenzie, 1964), was used as a genotype control to evaluate the alkalinity tolerance of N24852. These two varieties were grown in a growth chamber (E-41HO, Percival, USA) with 60% relative humidity, 15 h light (50000 lx)/9 h darkness photoperiod and corresponding temperature regime of 28°C/24°C. These two varieties showed obvious phenotypic difference when using 90 mM NaHCO_3_ treatment, but only showed subtle difference at 80 mM NaHCO_3_ and no difference at 100 mM NaHCO_3_ treatment in the preliminary experiment (Supplementary Figure S1). Therefore, 90 mM NaHCO_3_ was used for RNA-seq study.

Soybean seeds were surface-sterilized with 1% sodium hypochlorite for 30 s, and rinsed three times with deionized water. Twenty seeds were sown in plastic pots (Φ10 × 8 cm) which filled with clean quartz sand. Seven days after germination, the seedlings were thinned to four plants per pot. Four pots were placed in a plastic container (34 cm × 24 cm × 8 cm) containing 1.5 L fresh 1/2 strength Hoagland nutrient solution (pH ≈ 6.5), which could be absorbed through small holes at the bottom, and the solution was changed every 2 days. When the second trifoliolate leaves appeared (V3, approximately 14 days after planting), a treatment solution containing 90 mM NaHCO_3_ (with a final pH of 8.5, adjusted by addition of KOH) was applied to induce alkaline stress. The control contained no NaHCO_3_. The experiment included three independent biological replications.

### Tissue Harvest

Before harvest, roots were dipped into an iso-osmotic solution of 10 mM Ca(NO_3_)_2_ for 10–20 s to avoid turgor loss and rapid eﬄux of ions from the apoplast and epidermal cells due to osmotic shock (Munns et al., 2010), and rinsed three times with deionized water. Root tips (3 cm) of NaHCO_3_-treated and control plants were harvested after 12 and 24 h treatment, then immediately frozen in liquid nitrogen and stored at -80°C for RNA isolation. The remaining root tissues and leaves from the stressed and control plants were harvested at the same time points for measurement of ion concentration. For determination of ion concentrations, samples were pretreated at 105°C for 30 min to deactivate enzymes and dried at 80°C for 5 days. In order to minimize variation between individual plants, bulked roots and leaves were harvested from four uniform plants in a single pot for each biological replicate.

### Measurement of Ion Concentrations

Methods for ion extraction and measurement followed Munns et al. (2010). Dried samples were ground into powder. Then 0.1 g of the powder was digested in 2 mL nitric acid (HNO_3_) in a microwave digestion system (ETHOS T, Milestone, Italy) following the manufacturer’s recommendations. Na^+^, K^+^, and Ca^2+^ concentrations were estimated using an Inductively Coupled Plasma Optical Emission Spectrometer (ICP-OES) Optima 8000 (PerkinElmer, USA) according to the manufacturer’s instructions. The mg/L values were converted to mg/g dry weight (DW) using the formula of (concentration in mg/L as measured × dilution factor × volume of dilute nitrate acid)/(DW of tissue used in extraction × 1000). Differences between two soybean genotypes were compared by t-tests using the SAS proc ttest.

### RNA Isolation, cDNA Library Construction and RNA-seq

Roots of wild soybean N24852 under 90 mM NaHCO_3_ and control at 12 and 24 h were harvested for RNA extraction and RNA-seq. A total of 12 samples (2 time points × 2 treatments × 3 biological replicates) were prepared for RNA-seq. Total RNA was extracted using Trizol^®^ reagent (Invitrogen, USA). Total concentration and quality of the RNA samples were determined by agarose gel electrophoresis and a NanoDrop 2000 Spectrophotometer (Thermo Fisher Scientific, USA). An Agilent 2100 Bioanalyzer RNA Nano chip (Agilent Technologies, USA) was used for accurate quantification.

Messenger RNA (mRNA) was isolated from the total RNA by magnetic beads coated with oligo (dT) using a Dynabeads mRNA DIRECT Kit (Invitrogen, USA). Approximately 5 μg of mRNA from each sample was prepared to construct a library. The mRNA was fragmented into small pieces with fragmentation buffer at 90°C. Purified mRNA fragments were reverse transcribed (Invitrogen, USA) with random hexamer adaptors to synthesize the first strand cDNA. Second strand cDNA was synthesized with RNaseH (Invitrogen, USA) and DNA polymerase I (Invitrogen, USA). The cDNA was cleaned using Agencourt Ampure XP SPRI beads (Beckman Coulter, USA). The synthesized cDNAs were appended with an ‘A’ base at the 3′-end and ligated with paired-end adaptors. Paired-end cDNA libraries were amplified using PCR, and fragments of 400–500 bp insertions were selected from 2% agarose gel electrophoresis separation and quantified using qPCR. The twelve cDNA libraries were sequenced on an Illumina HiSeq 2500 platform (Illumina Inc. USA) according to the manufacturer’s recommendations at Berry Genomics Company, Beijing, China.

### Sequencing Data Analysis

The sequence data (in FastQ format) have been submitted to the National Center for Biotechnology Information (NCBI) Sequence Read Archive (SRA) databases with the study accession SRP093892. The raw reads were cleaned by removing adaptor reads and low-quality reads (ambiguous sequences with ‘*N*’ percentage values ≤3 and the percentage of low-quality bases less than 3 is ≥50%) using an in-house script written in C. The clean data were used for subsequent data analysis. High-quality clean reads were mapped to the latest version (*G. max Wm82.a2.v1*) of soybean reference genome (Goodstein et al., 2012) downloaded from phytozome^1^, using Tophat2 (v2.0.13) software (Kim et al., 2013). Mismatches of less than or equal to 2 bp were allowed in mapping to reference sequences. Subsequently, the sequencing saturation of the library, coverage analysis of clean reads on reference genes, as well as the genomic distributions in CDS (exons), introns, and intergenic regions were analyzed.

### Identification of Differentially Expressed Genes (DEGs)

Gene expression levels were normalized to Fragments Per Kilobase of transcript per Million fragments mapped (FPKM) by Cuﬄinks v2.2.2 software (Trapnell et al., 2010). DEGs were identified by comparing the NaHCO_3_-treated and control samples at the same time point using the R package DESeq (Anders and Huber, 2010). The significant DEGs were identified using the false discovery rate (FDR) ≤ 0.01 and | log_2_FoldChange|≥ 1.

### Functional Annotation and Pathway Analysis

All mapped unigenes and new genes found in this study were compared against seven databases including National Center for Biotechnology Information (NCBI) non-redundant protein database (NR), Gene Ontology (GO), Clusters of Orthologous Groups of proteins (COG) (Tatusov et al., 2000), euKaryotic Ortholog Groups (KOG) (Koonin et al., 2004), reviewed protein sequence database (Swiss-Prot) (Apweiler et al., 2004), and Kyoto Encyclopedia of Genes and Genomes (KEGG) Ontology (KO) (Kanehisa et al., 2004) using BLASTn (v 2.2.26) software (Altschul et al., 1999) with an *E*-value cutoff at 10^-5^, and searched against the Protein family (Pfam) database (Finn et al., 2014) by hmmscan (v 3.0) software (Johnson et al., 2010). All genes were also BLAST against the Plant Transcription Factors Database v3.0 (Jin et al., 2014) with an *E*-value cutoff at 10^-5^. The WEGO software package (Ye et al., 2006) was used for describing GO functional classification of cellular component, molecular function and biological process. GO enrichment analyses of DEGs were performed using Singular Enrichment Analysis (SEA) method with *P* < 0.01 and FDR < 0.05 by agriGO (Du et al., 2010), and the newest soybean genome Wm82.a2.v1 was set as background. The hypergeometric Fisher exact test (*P* < 0.01) and Benjamini and Hochberg method (FDR < 0.05) was performed to detect statistically significant enrichment of KEGG pathway and transcription factors, in comparison with the whole soybean transcriptome as the background. DEGs were also blasted against the KOG databases^2^ for functional classification.

### Heat Map with Clustering Analysis

Heat map of the DEGs overlapped between two time points (12 and 24 h), were analyzed using heatmap.2 function of the R/Bioconductor package gplots with default options (Warnes, 2016). The gene expression levels were transformed by log_2_ (FPKM+1) using three biological replications.

### Quantitative Real-Time PCR (qRT-PCR)

To validate the RNA-seq gene expression patterns, 14 DEGs were selected and investigated by qRT-PCR using the same RNA samples for the RNA-seq library construction. We used the housekeeping gene *GmUKN1* (*Glyma.12G020500*) as an internal control to normalize the level of gene expression (Hu et al., 2009; Guan et al., 2014). Primer pairs were designed for qRT-PCR using the online website NCBI primer-BLAST^3^. Primer sequences and gene annotations are listed in Supplementary Table S1. First-strand cDNA was synthesized from 500 ng/10 μl total RNA by using a PrimeScript RT Reagent Kit (TaKaRa, Japan). qRT-PCRs were performed in a LightCycler^®^ 480 II (Roche, Germany) in a final volume of 25 μl containing 12.5 μl SYBR Premix Ex Taq II (Tli RNaseH Plus) (TaKaRa, Japan), 2 μl (200 ng) of cDNA, 1 μl (10 mM) of the forward and reverse primers, and 8.5 μl of ddH_2_O. PCR conditions were set at 95°C pre-denaturation for 3 min and followed by 40 cycles of 95°C denaturation for 15 s, 60°C annealing for 15 s, and 72°C extension for 15 s. Relative gene expression level was calculated using the 2^-ΔΔCT^ method (Livak and Schmittgen, 2001). All qRT-PCR were performed in three technical replicates. The correlation of RNA-seq data with qRT-PCR analysis was calculated using SAS (v9.3) proc corr based on log_2_ fold changes.

## Results

### Phenotypic and Physiological Responses of Wild Soybean N24852 to NaHCO_3_ Stress

**FIGURE 1:**
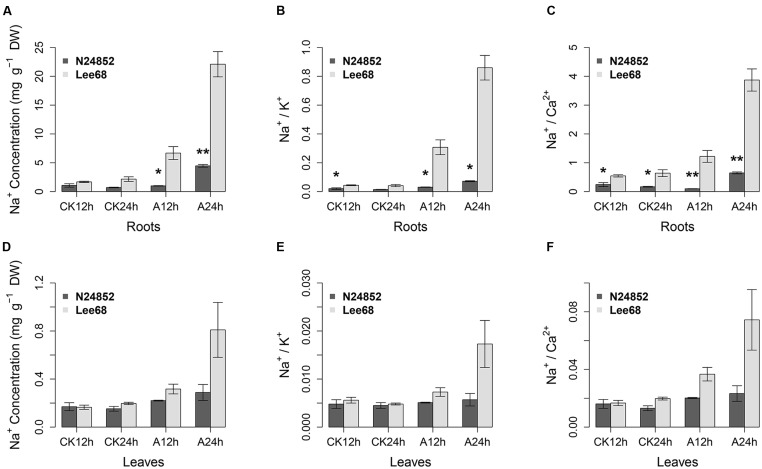
**Ion concentrations and ratios in N24852 and Lee 68 under control (CK) and alkaline stress for 12 and 24 h. (A)** Na^+^ concentration in roots, **(B)** Na^+^/K^+^ ratio in roots, **(C)** Na^+^/Ca^2+^ ratio in roots. **(D)** Na^+^ concentration in leaves, **(E)** Na^+^/K^+^ ratio in leaves, **(F)** Na^+^/Ca^2+^ ratio in leaves. Alkaline stress was 90 mM NaHCO_3_ (pH = 8.5) treatment, and control was 0 mM NaHCO_3_ (pH = 6.5). ^∗^ and ^∗∗^ indicate significant difference at 0.05 and 0.01 level by student’s *t*-tests between two soybean genotypes, respectively. DW, dry weight.

Maintenance of a low Na^+^ concentration, and low ratios of Na^+^/K^+^ and Na^+^/Ca^2+^ are widely used as indices of plant salinity tolerance (Puranik et al., 2011; Yong et al., 2014; Tuyen et al., 2016). To evaluate the alkalinity tolerance of wild soybean N24852, the salt tolerant soybean variety Lee 68 was used for comparison. The wild soybean N24852 showed more tolerance to 90 mM NaHCO_3_ stress than Lee 68, as indicated by the later appearance of chlorosis and wilting (Supplementary Figure S1). The concentration of Na^+^, ratios of Na^+^/K^+^ and Na^+^/Ca^2+^ in the roots of both soybean varieties increased after NaHCO_3_ treatment (**Figure 1**). But the wild soybean N24852 showed significantly (*P* < 0.05, student’s *t*-tests) lower concentration of Na^+^, ratios of Na^+^/K^+^ and Na^+^/Ca^2+^ in the roots than Lee68 under NaHCO_3_ stress (**Figure 1**). And the Na^+^ concentration, ratios of Na^+^/K^+^ and Na^+^/Ca^2+^ in the leaves of N24852 were also lower than those of Lee68 (**Figure 1**).

### RNA-seq Data Output, Quality Assessment, Mapping and Annotation

Due to the lower Na^+^ concentration in the roots of N24852 than that of Lee 68 (**Figure 1**) was observed as early as 12 h after alkalinity, RNA-seq analyses of N24852 roots after 12 and 24 h of NaHCO_3_ stress in comparison with control were performed on three independent biological replicates. A total of 12 cDNA libraries were constructed and sequenced on Illumina HiSeq 2500, and 283.6 million raw reads were generated (Supplementary Table S2). After removing adaptors and low quality sequences, a total of 270.5 million (95.38% of the raw reads) clean reads (approximately 67.63 Gb clean data) were obtained. On average, 23.6 million clean reads (5.64 Gb clean data) were obtained from each sample (Supplementary Table S2). The percentages of Phred-like quality scores at the Q30 level (an error probability of 1‰) ranged from 87.24 to 94.45% and the average GC content was estimated as 46.43% (Supplementary Table S2). Among the 12 samples, 88.16–91.69% of the clean reads were mapped to the reference genome, and 89.85–96.60% of clean reads were uniquely mapped (Supplementary Table S2). The saturation curves of 12 RNA-seq samples (genes with FPKM ≥ 0.01) estimated that the gene coverage started to show saturation when approximately more than 5 million clean reads were aligned (Supplementary Figure S2A). The average clean reads of our 12 samples were 22.54 million, which is more than the saturation threshold. Gene coverage analysis indicates that the sequencing reads were uniformly distributed from the 5′ to 3′ of genes (Supplementary Figure S2B). On average, more than 80% of the mapped reads were located at the exon region (Supplementary Figure S3). Detailed information of the RNA-seq data was listed in Supplementary Table S2, suggesting that the sequencing quality was high and sequencing depth was sufficient for transcriptome coverage.

A total of 54,844 unigenes were detected in our transcriptome by cuﬄinks program, including 54331 genes that were aligned to the soybean reference genome (96.94% of the total 56044 genes in Williams 82) and 513 new genes. Among these, 54342 genes (including 53888 mapped unigenes and 454 new genes) were annotated by at least one of the seven databases (Supplementary Table S3). Taken together, 96.08% (54342) of the expressed genes were successfully annotated in at least one of databases, with 10.51% (5946) of genes annotated in all databases (Supplementary Table S3). The 54342 annotated genes are listed in Supplementary Table S4.

### Identification of Differentially Expressed Genes (DEGs) in Soybean Roots under NaHCO_3_ Stress

**FIGURE 2:**
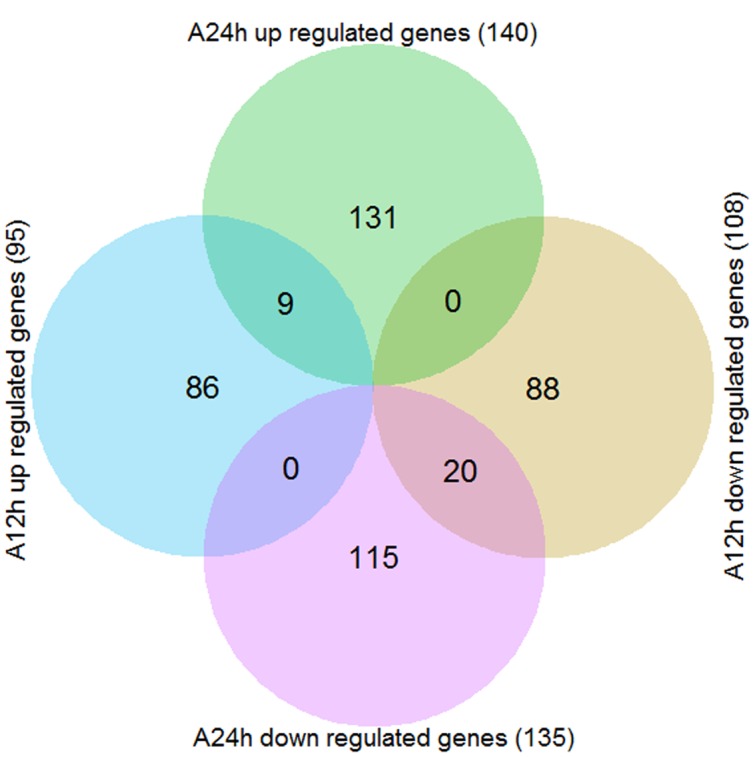
**Venn diagram of the differentially expressed genes (DEGs) between alkaline stress (90 mM NaHCO_3_, pH = 8.5) and control.** A12h, 12 h of alkaline stress; A24h, 24 h of alkaline stress.

**FIGURE 3:**
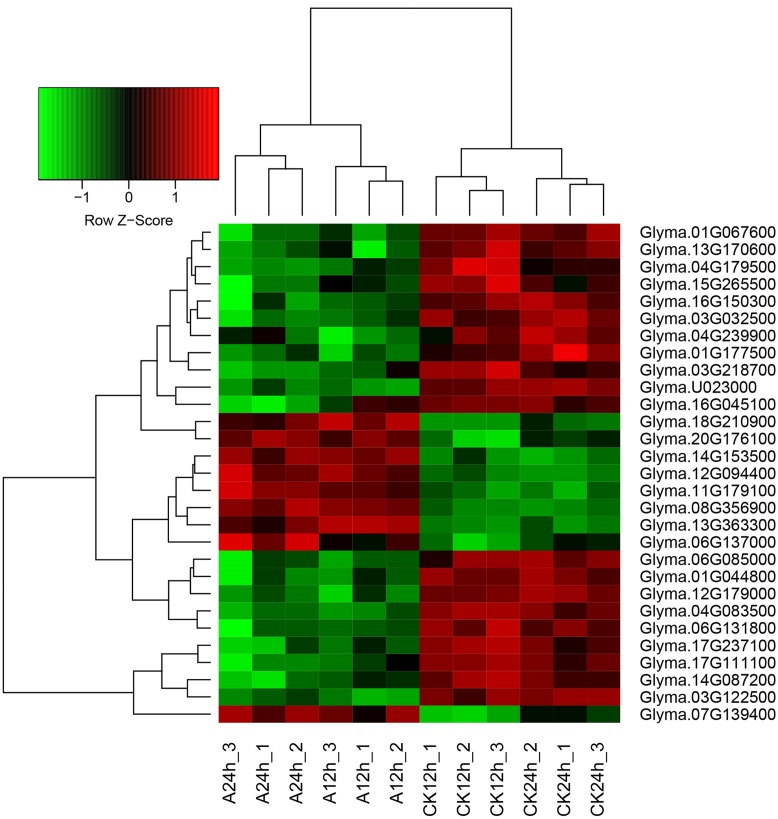
**Heatmap of the overlap DEGs between two time points (12 and 24 h).** Heatmap was plotted using heatmap.2 function of the R/Bioconductor package gplots. Hierarchical clustering of the DEGs was done by complete method with Euclidean distance. The gene expression levels were transformed by log_2_ (FPKM+1) and the values were centered and scaled in row direction. *X*-axis, samples; *Y*-axis, differentially expressed gene names.

By using the criteria of FDR ≤ 0.01 and | log_2_FoldChange|≥ 1, a total of 449 genes were differentially expressed in wild soybean roots under NaHCO_3_ stress compared with control (Supplementary Table S5). As shown in the Venn diagram (**Figure 2**), there were 95 and 140 up-regulated genes and 108 and 135 down-regulated genes after 12 and 24 h of alkaline stress, respectively, with more DEGs detected after 24 h alkaline stress than 12 h, implying more transcriptional changes at 24 h. The numbers of up- and down-regulated genes were similar. Nine genes were up-regulated and 20 genes were down-regulated at both time points (**Figures 2** and **3**), suggesting their importance in soybean response to NaHCO_3_ Stress. The overlapped up-regulated genes (**Figure 3**) included two aluminum-activated malate transporter (ALMT) genes (*Glyma.11G179100* and *Glyma.12G094400*) and one gene (*Glyma.13G363300*) encoding a late embryogenesis abundant protein (LEA). ALMT transporters have been reported to play roles in adaptation of plants to abiotic stress (Henderson et al., 2014), and LEA proteins accumulate in response to water deficit caused by drought, heat and salinity (Battaglia and Covarrubias, 2013; Kosova et al., 2014). No DEG showed opposite regulation patterns at two time points (**Figure 2**; Supplementary Table S5).

### Functional Classification and Gene Ontology (GO) Enrichment Analysis of DEGs

By WEGO database, the DEGs were classified into 12, 9, 12 categories for cellular components, molecular functions, and biological processes, respectively, after 12 h of NaHCO_3_ stress, and were classified into 12, 13, and 16 categories, respectively, at 24 h of NaHCO_3_ treatment (Supplementary Figure S4). By KOG database, there were 97 and 138 DEGs with KOG functional classification information at 12 and 24 h of NaHCO_3_ stress, respectively, and they were classified into 17 functional categories (Supplementary Figure S5).

**FIGURE 4:**
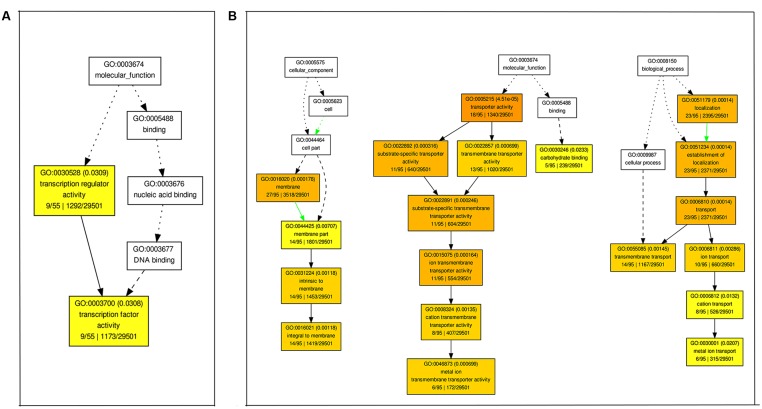
**Enriched Gene ontology (GO) terms for up-regulated genes at 12 h (A)** and 24 h **(B)** after 90 mM NaHCO_3_ (pH = 8.5) stress. The Singular Enrichment Analysis (SEA) was carried out by agriGO. The GO terms with their IDs are written in boxes. The significant (*P* < 0.01, FDR < 0.05) GO terms are in colored boxes (the degree of color saturation is positively correlated to the enrichment level of the GO term), and non-significant terms are in white boxes.

We also performed GO enrichment analyses of DEGs using agriGO (*P* < 0.01, FDR < 0.05). At 12 h of alkaline stress, genes with transcription factor activity (GO:0003700) were significantly enriched in up-regulated genes (**Figure 4A**), while genes with peptidase activity (GO:0008233) and related to biological process of proteolysis (GO:0006508) were enriched in down-regulated genes (Supplementary Figure S6A). At 24 h of alkaline stress, genes with the molecular function of transporter activity (GO:0005215) and carbohydrate binding (GO:0030246), and involved in biological process of transport (GO:0006810) were significantly enriched in up-regulated genes (**Figure 4B**), while GO terms in molecular functions of “hydrolase activity (GO:0016787),” “transferase activity (GO:0016757),” and “oxidoreductase activity (GO:0016705),” biological process of “carbohydrate metabolic process (GO:0005975)” especially “cellular glucan metabolic process (GO:0006073)” were enriched in down-regulated genes (Supplementary Figure S6B). Consistent with the number of DEGs, the numbers of enriched GO terms at 24 h were greater than 12 h after alkaline stress.

### KEGG Pathway Classification and Enrichment Analysis of DEGs

All DEGs were BLAST against the KEGG Ontology (KO) database. At 12 h of NaHCO_3_ stress, 30 DEGs were classified into 29 biological pathways belonging to 13 KEGG categories (Supplementary Table S6). At 24 h after NaHCO_3_ stress, 48 DEGs were classified into 37 biological pathways belonging to 12 KEGG categories (Supplementary Table S7).

We also used the Fisher exact test to analyze KEGG enrichment for DEGs (*P* < 0.01, FDR < 0.05). No enriched biological pathways were found at 12 h after NaHCO_3_ stress, but enrichment of “phenylpropanoid biosynthesis” and “phenylalanine metabolism” was found at 24 h after 90 mM NaHCO_3_ (pH = 8.5) treatment (Supplementary Figures S7 and S8). The genes involved in both pathways were peroxidase-encoding genes, including three up-regulated genes (*Glyma.07G209900*, *Glyma.17G053000*, and *Glyma.20G169200*) and one down-regulated gene (*Glyma.02G171600*). As one of the important antioxidant enzymes, peroxidases (PODs) could protect plants from oxidative damage by scavenging of reactive oxygen species (ROS), which is induced by various abiotic and biotic stresses (Miller et al., 2008; Gill and Tuteja, 2010).

### Validation of RNA-seq Data by qRT-PCR Analysis

**FIGURE 5:**
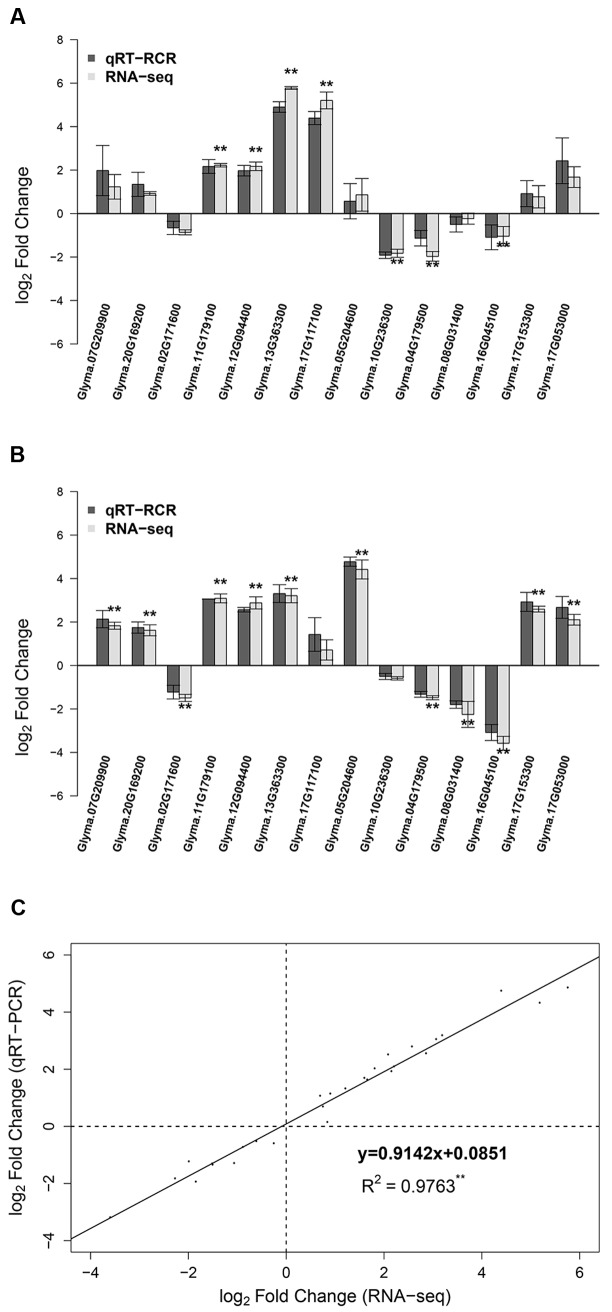
**Comparison of RNA-seq results and qRT-PCR analysis of gene expression levels. (A)** Log_2_ fold change of 14 genes at 12 h after 90 mM NaHCO_3_ (pH = 8.5) stress. **(B)** Log_2_ fold change of 14 genes at 24 h after 90 mM NaHCO_3_ (pH = 8.5) stress. **(C)** The comparison of log_2_ fold change obtained by RNA-seq (*x*-axis) and qRT-PCR (*y*-axis). ^∗∗^Differentially expressed genes at FDR ≤ 0.01 and | log_2_FoldChange|≥ 1 from RNA-seq.

To validate the expression data of RNA-seq, we selected 14 DEGs for qRT-PCR analysis, including four POD-encoding genes, three up-regulated genes and two down-regulated genes at both 12 and 24 h, and five genes that were up- or down- regulated at only one time point (**Figure 5**; Supplementary Table S1). To compare the expression data between RNA-seq and qRT-PCR, the relative expression level was transformed to log_2_ fold change. The qRT-PCR results showed a high consistency (linear regression equation *y* = 0.9142x + 0.0851, *R*^2^ = 0.9763) with RNA-seq data (**Figure 5**), indicating the reliability of RNA-seq expression profile in this study.

### DEGs Related to Transcription Factors

**FIGURE 6:**
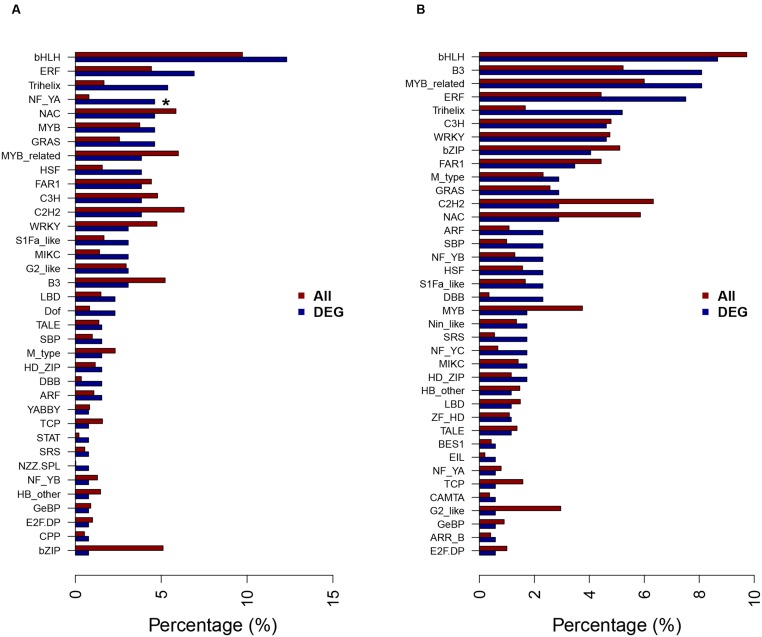
**Transcription factor families of the DEGs at 12 h (A)** and 24 h **(B)** of NaHCO_3_ treatment. A total of 130 and 173 transcription factors (TFs) were differentially expressed at 12 and 24 h after 90 mM NaHCO_3_ (pH 8.5) stress, respectively, and the percentages of TF families in DEGs and all genes in soybean genome were shown as blue bars and red bars, respectively. ^∗^Significantly enriched TF family identified by hypergeometric Fisher exact test (*P* < 0.01) and Benjamini and Hochberg method (FDR < 0.05).

Transcription factors are essential for regulation of gene expression, by binding to the specific *cis*-acting elements in the genes that they regulate. A total of 130 and 173 transcription factors representing 36 and 38 different families were differentially expressed at 12 and 24 h, respectively, under NaHCO_3_ stress comparing with control in wild soybean roots (**Figure 6**). Among the differentially expressed transcription factors, high percentages of basic helix-loop-helix (bHLH), ethylene-responsive factor (ERF), Trihelix, and zinc finger (C2H2, C3H) families were found at both time points. Nuclear Factor Y subunit A (NF-YA) family was enriched at 12 h after NaHCO_3_ stress. These transcription factors have already been reported to play roles in plant response to abiotic stress (Ge et al., 2010; Kielbowicz-Matuk, 2012; Li et al., 2013; Yong et al., 2014; Dey and Vlot, 2015). No transcription factor family was enriched at 24 h after NaHCO_3_ stress.

### DEGs Related to Ion Transport

ABC transporter (Lee et al., 2004; Henderson et al., 2014), ALMT family (Barbier-Brygoo et al., 2011; Henderson et al., 2014), glutamate receptor (GLR) (Demidchik et al., 2002), nitrate transporter (NRT)/proton dependent oligopeptide (POT) family (Li et al., 2010; Barbier-Brygoo et al., 2011; Chen et al., 2012), and S-type anion channel (SLAH) (Negi et al., 2008; Barbier-Brygoo et al., 2011) have been reported to play roles in ion transport, which indicates their importance in maintaining ion homeostasis in plant response to NaHCO_3_ stress. Nine transporter genes were differentially expressed at 12 h after NaHCO_3_ stress (**Table 1**). At 24 h after NaHCO_3_ treatment, the up-regulated genes were enriched in transport related genes as shown by GO enrichment analysis (**Figure 4B**). Twenty-four transporter genes were up-regulated and three transporter genes were down-regulated at 24 h after NaHCO_3_ stress (**Table 1**), including the genes encoding ABC transporter, ALMT, ammonium transporter, aquaporin transporter, cation eﬄux family, GLR, NRT/POT family, SLAH, sulfate transporter, and zinc/iron transporter. Our results indicate the importance of these transporter genes in maintaining ion homeostasis in soybean response to NaHCO_3_ stress.

**Table 1:** Transport related differentially expressed genes between NaHCO_3_ treatment and control in the roots of wild soybean N24852.

Time	Gene ID (Gmax 2.0)	Arabidopsis homolog (TAIR10)	Gene annotation	FDR	log_2_ FC
12 h	*Glyma.11G179100*	*AT1G08440.1*	Aluminum activated malate transporter (ALMT)	2.01E-07	2.23
	*Glyma.12G094400*	*AT1G08440.1*	Aluminum activated malate transporter (ALMT)	3.68E-05	2.17
	*Glyma.11G235200*	*AT2G40460.1*	NRT/POT Family	6.28E-04	1.95
	*Glyma.12G001700*	*AT1G22540.1*	NRT/POT Family	3.58E-03	-1.01
	*Glyma.03G122500*	*AT1G68570.1*	NRT/POT Family	9.92E-05	-1.14
	*Glyma.08G284000*	*AT1G12940.1*	NRT2.5/POT Family	7.48E-03	-1.19
	*Glyma.16G212900*	*AT1G65730.1*	Oligopeptide transporter (OPT)	2.08E-06	-1.29
	*Glyma.06G131800*	*AT4G27730.1*	Oligopeptide transporter (OPT)	1.93E-03	-1.78
	*Glyma.03G156700*	*AT3G51895.1*	Sulfate transporter	9.49E-04	-1.08
24 h	*Glyma.10G205600*	*AT3G25620.2*	ABC transporter	6.35E-03	1.57
	*Glyma.10G055000*	*AT2G36910.1*	ABC transporter	6.15E-04	1.18
	*Glyma.20G174800*	*AT1G71960.1*	ABC transporter	3.93E-03	1.12
	*Glyma.11G179100*	*AT1G08440.1*	Aluminum activated malate transporter (ALMT)	4.71E-09	3.09
	*Glyma.12G094400*	*AT1G08440.1*	Aluminum activated malate transporter (ALMT)	3.26E-04	2.88
	*Glyma.20G216800*	*AT4G00910.1*	Aluminum activated malate transporter (ALMT)	7.77E-05	1.80
	*Glyma.01G123400*	*AT2G38290.1*	Ammonium transporter	4.91E-08	1.67
	*Glyma.18G259500*	*AT5G37820.1*	Aquaporin transporter	9.67E-04	1.16
	*Glyma.05G208700*	*AT4G00430.1*	Aquaporin transporter	3.70E-04	-1.29
	*Glyma.09G122600*	*AT1G16310.1*	Cation eﬄux family	7.25E-04	1.60
	*Glyma.13G093300*	*AT2G29110.1*	Glutamate receptor (GLR)	2.07E-04	2.48
	*Glyma.17G153300*	*AT1G32450.1*	NRT/POT family	3.14E-19	2.59
	*Glyma.05G070600*	*AT1G32450.1*	NRT/POT family	1.47E-07	2.31
	*Glyma.01G200100*	*AT1G32450.1*	NRT/POT family	2.58E-03	1.69
	*Glyma.03G165900*	*AT5G46050.1*	NRT/POT family	4.15E-05	1.59
	*Glyma.03G122500*	*AT1G68570.1*	NRT/POT Family	8.29E-03	-1.03
	*Glyma.06G131800*	*AT4G27730.1*	Oligopeptide transporter (OPT)	1.15E-03	-2.08
	*Glyma.11G238400*	*AT5G10180.1*	Sulfate transporter	2.37E-03	2.24
	*Glyma.13G360000*	*AT3G15990.1*	Sulfate transporter	6.30E-04	1.48
	*Glyma.16G173600*	*AT5G24030.1*	S-type anion channel (SLAH)	2.28E-03	2.22
	*Glyma.10G229300*	*AT5G24030.1*	S-type anion channel (SLAH)	3.17E-05	1.39
	*Glyma.20G063100*	*AT3G12750.1*	Zinc/Iron transporter	1.11E-03	2.86
	*Glyma.15G262800*	*AT3G12750.1*	Zinc/Iron transporter	5.80E-14	2.29
	*Glyma.13G004400*	*AT3G12750.1*	Zinc/Iron transporter	7.72E-10	2.04
	*Glyma.20G022500*	*AT1G31260.1*	Zinc/Iron transporter	9.98E-07	1.85
	*Glyma.08G164400*	*AT3G12750.1*	Zinc/Iron transporter	1.19E-06	1.84
	*Glyma.06G052000*	*AT1G60960.1*	Zinc/Iron transporter	6.98E-06	1.83

*FC, fold change*.

## Discussion

The alkalinity-tolerant wild soybean variety N24852 showed lower Na^+^ concentration, and lower ratios of Na^+^/K^+^ and Na^+^/Ca^2+^ in leaves and roots under NaHCO_3_ stress in this study. This was similar to the previously reported salt tolerance phenotype of *G. max* (Tuyen et al., 2016), *Brassica napus* line N119 (Yong et al., 2014), *Oryza sativa* line FL478 (Walia et al., 2005), and *Foxtail millet* cv. Prasad (Puranik et al., 2011), all of which maintained lower Na^+^ concentrations, and ratios of Na^+^/K^+^ and Na^+^/Ca^2+^ compared with sensitive lines. For RNA sequencing, we used clean quartz as the cultivation medium to mimic the alkalinity in fields, and set up controls at each time point to identify DEGs between NaHCO_3_ stress and control, avoiding interference from the development-related DEGs.

Gene Ontology enrichment analyses of the DEGs showed that different GO terms were enriched for different time points. For example, genes with transcription factor activity (GO: 0003700) were significantly enriched in up-regulated genes at 12 h of alkaline stress (**Figure 4A**), while genes with transporter activity (GO: 0005215) were significantly enriched in up-regulated genes at 24 h of alkaline stress (**Figure 4B**). Transcription factors play important roles in plant response to abiotic stresses. NF-YA transcription factors were enriched at 12 h after NaHCO_3_ stress in this study (**Figure 6**). *AtNF-YA1* plays a role in regulating post-germination growth arrest under salt stress in Arabidopsis (Li et al., 2013). In addition, high percentages of bHLH, ERF, Trihelix, and zinc finger (C2H2, C3H) transcription factors were found at both 12 and 24 h after alkaline stress in our result (**Figure 6**). A great percentage of bHLH transcription factor family has been found in the transcriptome profiles of wild soybean roots (Ge et al., 2010) and leaves (Ge et al., 2011) under NaHCO_3_ treatment, and was enriched in the transcriptome of oilseed rape roots and leaves under NaCl treatment (Yong et al., 2014). AP2/ERF transcription factors have been shown to play roles in plant response to abiotic stress (Mizoi et al., 2012; Dey and Vlot, 2015) including salt stress (Wang L.Q. et al., 2015), and overexpression of *ERF1* in *Arabidopsis thaliana* enhanced plant tolerance to salt stress (Cheng et al., 2013). C2H2 zinc finger proteins are important components in regulation of plant tolerance to biotic and abiotic stresses (Kielbowicz-Matuk, 2012). Over-expression of a zinc finger protein gene *IbZFP1* from sweet potato increased salt and drought tolerance in transgenic Arabidopsis (Wang F. et al., 2015).

ABC transporters might play roles in Na^+^ and K^+^ homeostasis in Arabidopsis and Cl^-^ transport in grapevine (Lee et al., 2004; Henderson et al., 2014). Three ABC transporter genes were up-regulated at 24 h after NaHCO_3_ stress in this study (**Table 1**), suggesting their possible roles in mediating ion homeostasis of wild soybean under alkaline stress conditions. Among the nine up-regulated DEGs overlapped between the two time points, there are two *ALMT* genes. The *ALMT* gene family not only chelates aluminum in the plant rhizosphere, but also has multiple roles in plant adaptation to abiotic stress such as transport of anions involved in mineral nutrition and ion homeostasis processes (Henderson et al., 2014). Three *ALMT1* homolog genes changed expression levels in grapevine roots under 50 mM Cl^-^ conditions (Henderson et al., 2014). A gene (*Glyma.13G093300*) encoding GLR was up-regulated at 24 h after NaHCO_3_ stress (**Table 1**). GLR was predicted to have permeability to Na^+^ and K^+^, and regulated by Ca^2+^ (Demidchik et al., 2002). Four and five *NRT/POT* genes were differentially expressed at 12 and 24 h of NaHCO_3_ stress, respectively (**Table 1**). The NRT/POT family is involved in uptake of nitrate and various peptides (Tsay et al., 2007). *AtNRT1.8* was up-regulated while *AtNRT1.5* was down-regulated by salt and cadmium stress in Arabidopsis roots (Li et al., 2010; Chen et al., 2012). Two *SLAH* genes were up-regulated at 24 h of NaHCO_3_ stress, which encode the SLAHs and might be involved in the transmembrane transfer of anions (Barbier-Brygoo et al., 2011). The guard-cell-specific expression of *SLAC1/SLAH3* resulted in the dissipation of the over-accumulated osmoregulatory anions in the Arabidopsis *slac1* mutant (Negi et al., 2008).

KEGG enrichment analysis of DEGs showed “phenylpropanoid biosynthesis” and “phenylalanine metabolism” pathways were enriched at 24 h after NaHCO_3_ treatment (Supplementary Figures S7 and S8). A previous study using the Affymetrix^®^ Soybean Genome Array (Ge et al., 2010) indicated that genes participating in the “phenylpropanoid biosynthesis” pathway were significantly up-regulated in wild soybean roots under NaHCO_3_ stress. Transcriptomes of maize roots under Na_2_CO_3_ treatment (Zhang L.M. et al., 2013), oilseed rape roots under NaCl treatment (Yong et al., 2014), and bermudagrass roots under NaCl treatment (Hu et al., 2015) also showed an enrichment in the “phenylpropanoid biosynthesis” pathway. These results suggest “phenylpropanoid biosynthesis” pathway might play an important role in plant response to alkalinity and salinity. The DEGs involved in both “phenylpropanoid biosynthesis” and “phenylalanine metabolism” pathways in this study were POD genes, including three up-regulated (*Glyma.07G209900*, *Glyma.17G053000*, *Glyma.20G169200*) and one down-regulated (*Glyma.02G171600*). DuanMu et al. (2015) showed that two *POD* genes were differentially expressed in wild soybean roots treated with NaHCO_3_. As one of the important antioxidant enzymes, PODs could protect plants from oxidative damage by scavenging of ROS. Activities of POD in salt-tolerant species *Beta maritima* were higher than salt-sensitive relative *Beta vulgaris* at 150 and 500 mM NaCl stress (Chen et al., 2009). “Phenylpropanoid biosynthesis” pathway showed that POD also have an important role in lignin synthesis. Lignin is a phenylpropanoid compound derived from phenylalanine. Lignin and suberin could deposit in the primary cell wall and form into the Casparian strip in vascular plant roots, which appears as a tight barrier that reduces non-selective apoplastic transport of toxic solutes into the stelar tissues under alkaline-salt conditions (Chen et al., 2011).

Recently, great progress has been made on soybean tolerance to salinity. Several independent studies consistently verified a major quantitative trait locus (QTL) on Chromosome 3 for soybean tolerance to NaCl stress (Chen et al., 2008; Hamwieh and Xu, 2008; Hamwieh et al., 2011; Ha et al., 2013). A cation/H^+^ exchanger family gene, *GmCHX1/GmSALT3/Ncl/* (*Glyma03g32900*), was identified in this genomic region, which regulated Na^+^, K^+^, and Cl^-^ homeostasis in the shoot of soybean under NaCl stress, and the functional allele of this gene could improve soybean salt tolerance and yield under salinity stress (Guan et al., 2014; Qi et al., 2014; Tuyen et al., 2016). Guan et al. (2014) identified two salt-tolerant haplotypes and seven salt-sensitive haplotypes in *GmCHX1* by analyzing the SNP variation among 172 soybean accessions. Patil et al. (2016) identified three major structural variants and several SNPs in *GmCHX1* based on the re-sequencing (15X) of 129 soybean accessions.

The transcript abundance of *GmCHX1/GmSALT3/Ncl/Glyma03g32900* increased after NaCl treatment in salt-tolerant soybean lines (Guan et al., 2014; Qi et al., 2014). In the new version of soybean genome reference sequence (*Williams 82. a2.v1*), *Glyma03g32900* was assembled into two genes/transcript models, including *Glyma.03G171600* and *Glyma.03g171700* (Patil et al., 2016). In this study, these two genes/transcripts were not detected as a DEG at 12 or 24 h after 90 mM NaHCO_3_ treatment. And the published alkalinity-tolerance QTL were mapped on Chromosome 17 (Tuyen et al., 2010, 2013), which did not overlap with the major salt tolerance QTL on Chromosome 3 (Guan et al., 2014; Qi et al., 2014; Tuyen et al., 2016). These comparisons suggest that there might be different genes controlling soybean tolerance to alkalinity or salinity. The 68 candidate genes within the alkalinity-tolerance QTL on Chromosome 17 (Tuyen et al., 2013) were not detected as DEGs in this study, which might be due to different genes controlling alkalinity-tolerance in different soybean varieties (JWS156-1 for published QTL mapping study and N24852 for RNA-seq in this study). We did observe lower Na^+^ concentration, lower ratios of Na^+^/K^+^ and Na^+^/Ca^2+^ in both roots and leaves of the wild soybean variety N24852 under NaHCO_3_ stress (**Figure 1**), indicating that the alkalinity tolerance is primarily due to Na^+^ exclusion from the roots, which leads to less Na^+^ transport into the aerial part. In our analysis, genes related to ion transporters were enriched in NaHCO_3_ stress-responsive genes, which might play a role to maintain ion homeostasis of wild soybean (N24852) under alkalinity. Further studies, such as co-localization of alkalinity tolerance QTL with alkalinity-responsive genes using the same soybean tolerant variety, and functional study of candidate alkalinity-tolerance genes, would help to elucidate the genes and mechanisms underlying alkalinity tolerance.

On the other hand, under alkaline stress or salt stresses, plants encounter both ionic and osmotic stresses. Therefore, there might be some overlapped responsive genes between alkaline and salt stress, as well as other stresses. We compared the expression patterns of the alkalinity-responsive genes encoding NF-YA transcription factors and transporters in this study (Supplementary Table S8) with the published RNA-seq data in soybean under salt (Belamkar et al., 2014), drought (Belamkar et al., 2014; Chen et al., 2016), flooding (Chen et al., 2016), potassium deficiency (Wang et al., 2012), and shade stresses (Gong et al., 2014). Among these 39 alkalinity-responsive genes, there are 20, 16, 8, 9, and 8 genes were also detected as DEGs in soybean response to salt, drought, flooding, potassium deficiency, and shade, respectively. Therefore, some NF-YA transcription factors and transporters are involved in soybean response to general abiotic stresses mentioned above. More comprehensive analyses such as genome-wide comparisons of DEGs and GO and KEGG pathway enrichment could provide the overall picture of common and specific genes/pathways/mechanisms in soybean responses to different stresses, which would be a great study to follow in the future.

## Conclusion

In this study, an alkalinity-tolerant wild soybean variety N24852 was identified, which showed low Na^+^ concentration in both leaves and roots under NaHCO_3_ (pH = 8.5) stress. Nine genes (including two *ALMT* genes and one *LEA* gene) were up-regulated at both 12 and 24 h of NaHCO_3_ (pH = 8.5) treatment. NF-YA transcription factors were enriched in the up-regulated genes at 12 h after NaHCO_3_ stress, while genes related to ion transporters such as ABC transporter, ALMT, GLR, NRT/POT family, and SLAH were enriched in up-regulated genes at 24 h after NaHCO_3_ stress. “phenylpropanoid biosynthesis” and “phenylalanine metabolism” pathways were enriched at 24 h after NaHCO_3_ treatment. This study provides a list of NaHCO_3_ stress-responsive genes to help us further understanding plant response to alkaline stress.

## Author Contributions

JZ and YL conceived and designed the experiments. JZ, JW, and WJ performed the experiments. JZ, JW, WJ, JL, SY, and YL analyzed the data. JG and YL contributed reagents/materials and interpretation of the results. JZ and YL wrote and revised the manuscript. All authors read, revised and approved the final manuscript.

## Conflict of Interest Statement

The authors declare that the research was conducted in the absence of any commercial or financial relationships that could be construed as a potential conflict of interest.

## Footnotes

1 http://phytozome.jgi.doe.gov/pz/portal.html

2 http://www.ncbi.nlm.nih.gov/COG

3 http://www.ncbi.nlm.nih.gov/tools/primer-blast/index.cgi?LINK_LOC=BlastHome

## References

[B1] Abel G. H. Mackenzie A. J. (1964). Salt tolerance of soybean varieties (*Glycine max* L. Merrill) during germination and later growth. *Crop Sci.* 4 157–161. 10.2135/cropsci1964.0011183X000400020010x

[B2] Altschul S. Madden T. L. Schaffer A. A. Zhang J. Zhang Z. Miller W. (1999). Gapped BLAST and PSI-BLAST: a new generation of protein database search programs. *Nucleic Acids Res.* 1997 3389–3402. 10.1093/nar/25.17.3389 PMC146917 9254694

[B3] Anders S. Huber W. (2010). Differential expression analysis for sequence count data. *Genome Biol.* 11:R106 10.1186/Gb-2010-11-10-R106 PMC3218662 20979621

[B4] Apweiler R. Bairoch A. Wu C. H. Barker W. C. Boeckmann B. Ferro S. (2004). UniProt: the universal protein knowledgebase. *Nucleic Acids Res.* 32 D115–D119. 10.1093/nar/gkh131 14681372 PMC308865

[B5] Barbier-Brygoo H. De Angeli A. Filleur S. Frachisse J.-M. Gambale F. Thomine S. (2011). Anion channels/transporters in Plants: from molecular bases to regulatory networks. *Annu. Rev. Plant Biol.* 62 25–51. 10.1146/annurev-arplant-042110-103741 21275645

[B6] Battaglia M. Covarrubias A. A. (2013). Late embryogenesis abundant (LEA) proteins in legumes. *Front. Plant Sci.* 4:190 10.3389/Fpls.2013.00190 PMC3691520 23805145

[B7] Belamkar V. Weeks N. T. Bharti A. K. Farmer A. D. Graham M. A. Cannon S. B. (2014). Comprehensive characterization and RNA-Seq profiling of the HD-Zip transcription factor family in soybean (*Glycine max*) during dehydration and salt stress. *BMC Genomics* 15:950 10.1186/1471-2164-15-950 PMC4226900 25362847

[B8] Carter T. E. Nelson R. L. Sneller C. H. Cui Z. (2004). *Soybeans: Improvement, Production, and Uses: Agronomy Monograph*, 3rd Edn. Madison, WI: American Society of Agronomy, Crop Science Society of America, Soil Science Society of America.

[B9] Chen C. Z. Lv X. F. Li J. Y. Yi H. Y. Gong J. M. (2012). *Arabidopsis* NRT1.5 is another essential component in the regulation of nitrate reallocation and stress tolerance. *Plant Physiol.* 159 1582–1590. 10.1104/pp.112.199257 22685171 PMC3425198

[B10] Chen H. T. Cui S. Y. Fu S. X. Gai J. Y. Yu D. Y. (2008). Identification of quantitative trait loci associated with salt tolerance during seedling growth in soybean (*Glycine max* L.). *Aust. J. Agric. Res.* 59 1086–1091. 10.1071/AR08104

[B11] Chen T. Cai X. Wu X. Karahara I. Schreiber L. Lin J. (2011). Casparian strip development and its potential function in salt tolerance. *Plant Signal. Behav.* 6 1499–1502. 10.4161/psb.6.10.17054 21904117 PMC3256377

[B12] Chen W. Yao Q. Patil G. B. Agarwal G. Deshmukh R. K. Lin L. (2016). Identification and comparative analysis of differential gene expression in soybean leaf tissue under drought and flooding stress revealed by RNA-Seq. *Front. Plant Sci.* 7:1044 10.3389/fpls.2016.01044 PMC4950259 27486466

[B13] Chen W. C. Cui P. J. Sun H. Y. Guo W. Q. Yang C. W. Jin H. (2009). Comparative effects of salt and alkali stresses on organic acid accumulation and ionic balance of seabuckthorn (*Hippophae rhamnoides* L.). *Ind. Crops Prod.* 30 351–358. 10.1016/j.indcrop.2009.06.007

[B14] Cheng M. C. Liao P. M. Kuo W. W. Lin T. P. (2013). The *Arabidopsis* EthyleneResponse Factor1 regulates abiotic stress-responsive gene expression by binding to different cis-acting elements in response to different stress signals. *Plant Physiol.* 162 1566–1582. 10.1104/pp.113.221911 23719892 PMC3707555

[B15] Demidchik V. Davenport R. J. Tester M. (2002). Nonselective cation channels in plants. *Annu. Rev. Plant Biol.* 53 67–107. 10.1146/annurev.arplant.53.091901.161540 12221989

[B16] Dey S. Vlot A. C. (2015). Ethylene responsive factors in the orchestration of stress responses in monocotyledonous plants. *Front. Plant Sci.* 6:640 10.3389/Fpls.2015.00640 PMC4552142 26379679

[B17] Du Z. Zhou X. Ling Y. Zhang Z. H. Su Z. (2010). agriGO: a GO analysis toolkit for the agricultural community. *Nucleic Acids Res.* 38 W64–W70. 10.1093/nar/gkq310 20435677 PMC2896167

[B18] DuanMu H. Wang Y. Bai X. Cheng S. Deyholos M. K. Wong G. K. (2015). Wild soybean roots depend on specific transcription factors and oxidation reduction related genesin response to alkaline stress. *Funct. Integr. Genomics* 15 651–660. 10.1007/s10142-015-0439-y 25874911

[B19] Fan X. D. Wang J. Q. Yang N. Dong Y. Y. Liu L. Wang F. W. (2013). Gene expression profiling of soybean leaves and roots under salt, saline-alkali and drought stress by high-throughput Illumina sequencing. *Gene* 512 392–402. 10.1016/j.gene.2012.09.100 23063936

[B20] FAO (2000). *Extent and Causes of Salt Affected Soils in Participating Countries.* Available at: http://www.fao.org/ag/agl/agll/spush/topic2.htm

[B21] Finn R. D. Bateman A. Clements J. Coggill P. Eberhardt R. Y. Eddy S. R. (2014). Pfam: the protein families database. *Nucleic Acids Res.* 42 D222–D230. 10.1093/nar/gkt1223 24288371 PMC3965110

[B22] Gaxiola R. A. Li J. S. Undurraga S. Dang L. M. Allen G. J. Alper S. L. (2001). Drought- and salt-tolerant plants result from overexpression of the AVP1 H+-pump. *Proc. Natl. Acad. Sci. U.S.A.* 98 11444–11449. 10.1073/pnas.191389398 11572991 PMC58749

[B23] Gaxiola R. A. Rao R. Sherman A. Grisafi P. Alper S. L. Fink G. R. (1999). The *Arabidopsis thaliana* proton transporters, AtNhx1 and Avp1, can function in cation detoxification in yeast. *Proc. Natl. Acad. Sci. U.S.A.* 96 1480–1485. 10.1073/pnas.96.4.1480 9990049 PMC15488

[B24] Ge Y. Li Y. Lv D. K. Bai X. Ji W. Cai H. (2011). Alkaline-stress response in *Glycine soja* leaf identifies specific transcription factors and ABA-mediated signaling factors. *Funct. Integr. Genomics* 11 369–379. 10.1007/s10142-010-0191-2 20938706

[B25] Ge Y. Li Y. Zhu Y. M. Bai X. Lv D. K. Guo D. (2010). Global transcriptome profiling of wild soybean (*Glycine soja*) roots under NaHCO3 treatment. *BMC Plant Biol.* 10:153 10.1186/1471-2229-10-153 PMC3017823 20653984

[B26] Gill S. S. Tuteja N. (2010). Reactive oxygen species and antioxidant machinery in abiotic stress tolerance in crop plants. *Plant Physiol. Biochem.* 48 909–930. 10.1016/j.plaphy.2010.08.016 20870416

[B27] Gong W. Qi P. Du J. Sun X. Wu X. Song C. (2014). Transcriptome analysis of shade-induced inhibition on leaf size in relay intercropped soybean. *PLoS ONE* 9:e98465 10.1371/journal.pone.0098465 PMC4041726 24886785

[B28] Goodstein D. M. Shu S. Q. Howson R. Neupane R. Hayes R. D. Fazo J. (2012). Phytozome: a comparative platform for green plant genomics. *Nucleic Acids Res.* 40 D1178–D1186. 10.1093/nar/gkr944 22110026 PMC3245001

[B29] Guan R. Qu Y. Guo Y. Yu L. Liu Y. Jiang J. (2014). Salinity tolerance in soybean is modulated by natural variation in GmSALT3. *Plant J.* 80 937–950. 10.1111/tpj.12695 25292417

[B30] Ha B. K. Vuong T. D. Velusamy V. Nguyen H. T. Shannon J. G. Lee J. D. (2013). Genetic mapping of quantitative trait loci conditioning salt tolerance in wild soybean (*Glycine soja*) PI 483463. *Euphytica* 193 79–88. 10.1007/s10681-013-0944-9

[B31] Hamwieh A. Tuyen D. D. Cong H. Benitez E. R. Takahashi R. Xu D. H. (2011). Identification and validation of a major QTL for salt tolerance in soybean. *Euphytica* 179 451–459. 10.1007/s10681-011-0347-8

[B32] Hamwieh A. Xu D. H. (2008). Conserved salt tolerance quantitative trait locus (QTL) in wild and cultivated soybeans. *Breed. Sci.* 58 355–359. 10.1270/Jsbbs.58.355

[B33] Henderson S. W. Baumann U. Blackmore D. H. Walker A. R. Walker R. R. Gilliham M. (2014). Shoot chloride exclusion and salt tolerance in grapevine is associated with differential ion transporter expression in roots. *BMC Plant Biol.* 14:273 10.1186/S12870-014-0273-8 PMC4220414 25344057

[B34] Hu L. Li H. Chen L. Lou Y. Amombo E. Fu J. (2015). RNA-seq for gene identification and transcript profiling in relation to root growth of bermudagrass (*Cynodon dactylon*) under salinity stress. *BMC Genomics* 16:575 10.1186/s12864-015-1799-3 PMC4523028 26238595

[B35] Hu R. Fan C. Li H. Zhang Q. Fu Y. F. (2009). Evaluation of putative reference genes for gene expression normalization in soybean by quantitative real-time RT-PCR. *BMC Mol. Biol.* 10:93 10.1186/1471-2199-10-93 PMC2761916 19785741

[B36] Hyten D. L. Song Q. J. Zhu Y. L. Choi I. Y. Nelson R. L. Costa J. M. (2006). Impacts of genetic bottlenecks on soybean genome diversity. *Proc. Natl. Acad. Sci. U.S.A.* 103 16666–16671. 10.1073/pnas.0604379103 17068128 PMC1624862

[B37] Jin J. Zhang H. Kong L. Gao G. Luo J. (2014). PlantTFDB 3.0: a portal for the functional and evolutionary study of plant transcription factors. *Nucleic Acids Res.* 42 D1182–D1187. 10.1093/nar/gkt1016 24174544 PMC3965000

[B38] Johnson L. S. Eddy S. R. Portugaly E. (2010). Hidden Markov model speed heuristic and iterative HMM search procedure. *BMC Bioinformatics* 11:431 10.1186/1471-2105-11-431 PMC2931519 20718988

[B39] Kanehisa M. Goto S. Kawashima S. Okuno Y. Hattori M. (2004). The KEGG resource for deciphering the genome. *Nucleic Acids Res.* 32 D277–D280. 10.1093/nar/gkh063 14681412 PMC308797

[B40] Kielbowicz-Matuk A. (2012). Involvement of plant C(2)H(2)-type zinc finger transcription factors in stress responses. *Plant Sci.* 18 78–85. 10.1016/j.plantsci.2011.11.015 22325868

[B41] Kim D. Pertea G. Trapnell C. Pimentel H. Kelley R. Salzberg S. L. (2013). TopHat2: accurate alignment of transcriptomes in the presence of insertions, deletions and gene fusions. *Genome Biol.* 14:R36 10.1186/Gb-2013-14-4-R36 PMC4053844 23618408

[B42] Kim M. Y. Lee S. Van K. Kim T. H. Jeong S. C. Choi I. Y. (2010). Whole-genome sequencing and intensive analysis of the undomesticated soybean (*Glycine soja* Sieb. and Zucc.) genome. *Proc. Natl. Acad. Sci. U.S.A.* 107 22032–22037. 10.1073/pnas.1009526107 21131573 PMC3009785

[B43] Koonin E. V. Fedorova N. D. Jackson J. D. Jacobs A. R. Krylov D. M. Makarova K. S. (2004). A comprehensive evolutionary classification of proteins encoded in complete eukaryotic genomes. *Genome Biol.* 5:R7 10.1186/Gb-2004-5-2-R7 PMC395751 14759257

[B44] Korir P. C. Zhang J. Wu K. Zhao T. Gai J. (2013). Association mapping combined with linkage analysis for aluminum tolerance among soybean cultivars released in Yellow and Changjiang River Valleys in China. *Theor. Appl. Genet.* 126 1659–1675. 10.1007/s00122-013-2082-0 23515677

[B45] Kosova K. Vitamvas P. Prasil I. T. (2014). Wheat and barley dehydrins under cold, drought, and salinity - what can LEA-II proteins tell us about plant stress response? *Front. Plant Sci.* 5:343 10.3389/Fpls.2014.00343 PMC4089117 25071816

[B46] Krishnamurthy P. Lee J. D. Ha B. K. Chae J. H. Song J. T. Tsukamoto C. (2015). Genetic characterization of group A acetylsaponin-deficient mutants from wild soybean (*Glycine soja* Sieb. and Zucc.). *Plant Breed.* 134 316–321. 10.1111/pbr.12269

[B47] Lam H. M. Xu X. Liu X. Chen W. B. Yang G. H. Wong F. L. (2010). Resequencing of 31 wild and cultivated soybean genomes identifies patterns of genetic diversity and selection. *Nat. Genet.* 42 1053–1059. 10.1038/ng.715 21076406

[B48] Lee E. K. Kwon M. Ko J. H. Yi H. Hwang M. G. Chang S. (2004). Binding of sulfonylurea by AtMRP5, an *Arabidopsis* multidrug resistance-related protein that functions in salt tolerance. *Plant Physiol.* 134 528–538. 10.1104/pp.103.027045 14684837 PMC316332

[B49] Li J. Y. Fu Y. L. Pike S. M. Bao J. Tian W. Zhang Y. (2010). The *Arabidopsis* nitrate transporter NRT1.8 functions in nitrate removal from the xylem sap and mediates cadmium tolerance. *Plant Cell* 22 1633–1646. 10.1105/tpc.110.075242 20501909 PMC2899866

[B50] Li Y. H. Zhou G. Ma J. Jiang W. Jin L. G. Zhang Z. (2014). De novo assembly of soybean wild relatives for pan-genome analysis of diversity and agronomic traits. *Nat. Biotechnol.* 32 1045–1052. 10.1038/nbt.2979 25218520

[B51] Li Y. J. Fang Y. Fu Y. R. Huang J. G. Wu C. A. Zheng C. C. (2013). NFYA1 is involved in regulation of postgermination growth arrest under salt stress in *Arabidopsis*. *PLoS ONE* 8:e61289 10.1371/journal.pone.0061289 PMC3634844 23637805

[B52] Livak K. J. Schmittgen T. D. (2001). Analysis of relative gene expression data using real-time quantitative PCR and the 2(T)(-Delta Delta C) method. *Methods* 25 402–408. 10.1006/meth.2001.1262 11846609

[B53] Miller G. Shulaev V. Mittler R. (2008). Reactive oxygen signaling and abiotic stress. *Physiol. Plant.* 133 481–489. 10.1111/j.1399-3054.2008.01090.x 18346071

[B54] Mizoi J. Shinozaki K. Yamaguchi-Shinozaki K. (2012). AP2/ERF family transcription factors in plant abiotic stress responses. *Biochim. Biophys. Acta* 1819 86–96. 10.1016/j.bbagrm.2011.08.004 21867785

[B55] Munns R. (2005). Genes and salt tolerance: bringing them together. *New Phytol.* 167 645–663. 10.1111/j.1469-8137.2005.01487.x 16101905

[B56] Munns R. Tester M. (2008). Mechanisms of salinity tolerance. *Annu. Rev. Plant Biol.* 59 651–681. 10.1146/annurev.arplant.59.032607.092911 18444910

[B57] Munns R. Wallace P. A. Teakle N. L. Colmer T. D. (2010). Measuring soluble ion concentrations (Na(+), K(+), Cl(-)) in salt-treated plants. *Methods Mol. Biol.* 639 371–382. 10.1007/978-1-60761-702-0_23 20387059

[B58] Negi J. Matsuda O. Nagasawa T. Oba Y. Takahashi H. Kawai-Yamada M. (2008). CO2 regulator SLAC1 and its homologues are essential for anion homeostasis in plant cells. *Nature* 452 483–486. 10.1038/nature06720 18305482

[B59] Pardo J. M. Cubero B. Leidi E. O. Quintero F. J. (2006). Alkali cation exchangers: roles in cellular homeostasis and stress tolerance. *J. Exp. Bot.* 57 1181–1199. 10.1093/jxb/erj114 16513813

[B60] Patil G. Do T. Vuong T. D. Valliyodan B. Lee J. D. Chaudhary J. (2016). Genomic-assisted haplotype analysis and the development of high-throughput SNP markers for salinity tolerance in soybean. *Sci. Rep.* 6:19199 10.1038/srep19199 PMC4726057 26781337

[B61] Peiffer G. A. King K. E. Severin A. J. May G. D. Cianzio S. R. Lin S. F. (2012). Identification of candidate genes underlying an iron efficiency quantitative trait locus in soybean. *Plant Physiol.* 158 1745–1754. 10.1104/pp.111.189860 22319075 PMC3320182

[B62] Puranik S. Jha S. Srivastava P. S. Sreenivasulu N. Prasad M. (2011). Comparative transcriptome analysis of contrasting foxtail millet cultivars in response to short-term salinity stress. *J. Plant Physiol.* 168 280–287. 10.1016/j.jplph.2010.07.005 20708821

[B63] Qi X. Li M. W. Xie M. Liu X. Ni M. Shao G. (2014). Identification of a novel salt tolerance gene in wild soybean by whole-genome sequencing. *Nat. Commun.* 5:4340 10.1038/ncomms5340 PMC4104456 25004933

[B64] Schmutz J. Cannon S. B. Schlueter J. Ma J. Mitros T. Nelson W. (2010). Genome sequence of the palaeopolyploid soybean. *Nature* 463 178–183. 10.1038/nature08670 20075913

[B65] Serrano R. Rodriguez-Navarro A. (2001). Ion homeostasis during salt stress in plants. *Curr. Opin. Cell Biol.* 13 399–404. 10.1016/S0955-0674(00)00227-1 11454443

[B66] Tatusov R. L. Galperin M. Y. Natale D. A. Koonin E. V. (2000). The COG database: a tool for genome-scale analysis of protein functions and evolution. *Nucleic Acids Res.* 28 33–36. 10.1093/Nar/28.1.33 10592175 PMC102395

[B67] Trapnell C. Williams B. A. Pertea G. Mortazavi A. Kwan G. van Baren M. J. (2010). Transcript assembly and quantification by RNA-Seq reveals unannotated transcripts and isoform switching during cell differentiation. *Nat. Biotechnol.* 28 511–515. 10.1038/nbt.1621 20436464 PMC3146043

[B68] Tsay Y. F. Chiu C. C. Tsai C. B. Ho C. H. Hsu P. K. (2007). Nitrate transporters and peptide transporters. *FEBS Lett.* 581 2290–2300. 10.1016/j.febslet.2007.04.047 17481610

[B69] Tuyen D. D. Chen H. T. Vu H. T. T. Hamwieh A. Yamada T. Sato T. (2016). Ncl synchronously regulates Na+, K+, and Cl- in soybean and greatly increases the grain yield in saline field conditions. *Sci. Rep.* 6:19147 10.1038/srep19147 PMC4705485 26744076

[B70] Tuyen D. D. Lal S. K. Xu D. H. (2010). Identification of a major QTL allele from wild soybean (*Glycine soja* Sieb. & Zucc.) for increasing alkaline salt tolerance in soybean. *Theor. Appl. Genet.* 121 229–236. 10.1007/s00122-010-1304-y 20204319

[B71] Tuyen D. D. Zhang H. M. Xu D. H. (2013). Validation and high-resolution mapping of a major quantitative trait locus for alkaline salt tolerance in soybean using residual heterozygous line. *Mol. Breed.* 31 79–86. 10.1007/s11032-012-9771-2

[B72] Walia H. Wilson C. Condamine P. Liu X. Ismail A. M. Zeng L. H. (2005). Comparative transcriptional profiling of two contrasting rice genotypes under salinity stress during the vegetative growth stage. *Plant Physiol.* 139 822–835. 10.1104/pp.105.065961 16183841 PMC1255998

[B73] Wang C. Chen H. Hao Q. Sha A. Shan Z. Chen L. (2012). Transcript profile of the response of two soybean genotypes to potassium deficiency. *PLoS ONE* 7:e39856 10.1371/journal.pone.0039856 PMC3390323 22792192

[B74] Wang F. Tong W. Zhu H. Kong W. Peng R. Liu Q. (2015). A novel Cys/His zinc finger protein gene from sweetpotato, IbZFP1, is involved in salt and drought tolerance in transgenic *Arabidopsis*. *Planta* 243 783–797. 10.1007/s00425-015-2443-9 26691387

[B75] Wang L. Q. Wang C. Qin L. P. Liu W. J. Wang Y. C. (2015). ThERF1 regulates its target genes via binding to a novel cis-acting element in response to salt stress. *J. Integr. Plant Biol.* 57 838–847. 10.1111/jipb.12335 25641039

[B76] Wang W. B. He Q. Y. Yang H. Y. Xiang S. H. Zhao T. J. Gai J. Y. (2013). Development of a chromosome segment substitution line population with wild soybean (*Glycine soja* Sieb. et Zucc.) as donor parent. *Euphytica* 189 293–307. 10.1007/s10681-012-0817-7

[B77] Warnes G. R. (2016). *gplots: Various R Programming Tools for Plotting Data*. Available at: https://cran.r-project.org/web/packages/gplots/.

[B78] Xu D. Tuyen do D. (2012). Genetic studies on saline and sodic tolerances in soybean. *Breed. Sci.* 61 559–565. 10.1270/jsbbs.61.559 23136495 PMC3406780

[B79] Xu D. H. Gai J. Y. (2003). Genetic diversity of wild and cultivated soybeans growing in China revealed by RAPD analysis. *Plant Breed.* 122 503–506. 10.1046/j.0179-9541.2003.00911.x

[B80] Yang C. W. Xu H. H. Wang L. L. Liu J. Shi D. C. Wang D. L. (2009). Comparative effects of salt-stress and alkali-stress on the growth, photosynthesis, solute accumulation, and ion balance of barley plants. *Photosynthetica* 47 79–86. 10.1007/s11099-009-0013-8

[B81] Yao D. Zhang X. Zhao X. Liu C. Wang C. Zhang Z. (2011). Transcriptome analysis reveals salt-stress-regulated biological processes and key pathways in roots of cotton (*Gossypium hirsutum* L.). *Genomics* 98 47–55. 10.1016/j.ygeno.2011.04.007 21569837

[B82] Ye J. Fang L. Zheng H. K. Zhang Y. Chen J. Zhang Z. J. (2006). WEGO: a web tool for plotting GO annotations. *Nucleic Acids Res.* 34 W293–W297. 10.1093/nar/gkl1031 16845012 PMC1538768

[B83] Yong H. Y. Zou Z. Kok E. P. Kwan B. H. Chow K. Nasu S. (2014). Comparative transcriptome analysis of leaves and roots in response to sudden increase in salinity in Brassica napus by RNA-seq. *Biomed. Res. Int.* 2014:467395 10.1155/2014/467395 PMC4142189 25177691

[B84] Zhang L. M. Liu X. G. Qu X. N. Yu Y. Han S. P. Dou Y. (2013). Early transcriptomic adaptation to Na(2)CO(3) stress altered the expression of a quarter of the total genes in the maize genome and exhibited shared and distinctive profiles with NaCl and high pH stresses. *J. Integr. Plant Biol.* 55 1147–1165. 10.1111/jipb.12100 24034274

[B85] Zhang X. Wei L. Q. Wang Z. Z. Wang T. (2013). Physiological and molecular features of *Puccinellia tenuiflora* tolerating salt and alkaline-salt stress. *J. Integr. Plant Biol.* 55 262–276. 10.1111/jipb.12013 23176661

[B86] Zhang Y. Liu Z. Khan A. A. Lin Q. Han Y. Mu P. (2016). Expression partitioning of homeologs and tandem duplications contribute to salt tolerance in wheat (*Triticum aestivum* L.). *Sci. Rep.* 6:21476 10.1038/srep21476 PMC4759826 26892368

[B87] Zhou Y. Yang P. Cui F. Zhang F. Luo X. Xie J. (2016). Transcriptome analysis of salt stress responsiveness in the seedlings of Dongxiang wild rice (*Oryza rufipogon* Griff.). *PLoS ONE* 11:e0146242 10.1371/journal.pone.0146242 PMC4709063 26752408

[B88] Zhou Z. Jiang Y. Wang Z. Gou Z. Lyu J. Li W. (2015). Resequencing 302 wild and cultivated accessions identifies genes related to domestication and improvement in soybean. *Nat. Biotechnol.* 33 408–414. 10.1038/nbt.3096 25643055

[B89] Zhu J. K. (2001). Plant salt tolerance. *Trends Plant Sci.* 6 66–71. 10.1016/S1360-1385(00)01838-0 11173290

[B90] Zhu J. K. (2003). Regulation of ion homeostasis under salt stress. *Curr. Opin. Plant Biol.* 6 441–445. 10.1016/S1369-5266(03)00085-2 12972044

