# Supplementary material for: Identification and Analysis of NaHCO3 Stress Responsive Genes in Wild Soybean (Glycine soja) Roots by RNA-seq
Source: Front Plant Sci. 2016 Dec 9;7:1842. doi: 10.3389/fpls.2016.01842 (PMC5161042; doi:10.3389/fpls.2016.01842)
Supplement: Supplementary file 9 [file Image_1.PDF]

Supplementary Material

Identification and Analysis of $NaHCO_3$ Stress Responsive Genes in Wild Soybean (*Glycine soja*) Roots by RNA-seq

Jinlong Zhang[1], Jiaxue Wang[1], Wei Jiang[1], Juge Liu[1], Songnan Yang[1], Junyi Gai[1], Yan Li[1*]

[1]National Key Laboratory of Crop Genetics and Germplasm Enhancement, National Center for Soybean Improvement, Key Laboratory for Biology and Genetic Improvement of Soybean (General, Ministry of Agriculture), Jiangsu Collaborative Innovation Center for Modern Crop Production, Nanjing Agricultural University, Nanjing, China

[*] Correspondence:

Yan Li

yanli1@njau.edu.cn

Supplementary Figures

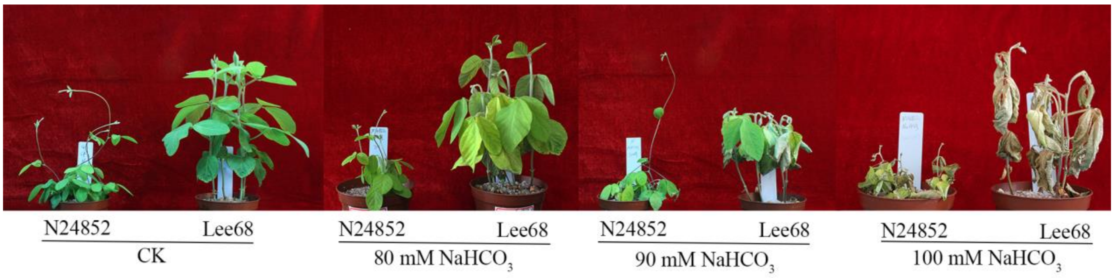

Figure S1 | Phenotypes of the wild soybean variety N24852 and cultivated soybean variety Lee 68 under alkaline stress for 5 days. CK: check, 0 mM $NaHCO_3$, pH ≈ 6.5; Alkaline stress: 80, 90, 100 mM $NaHCO_3$, pH = 8.5.

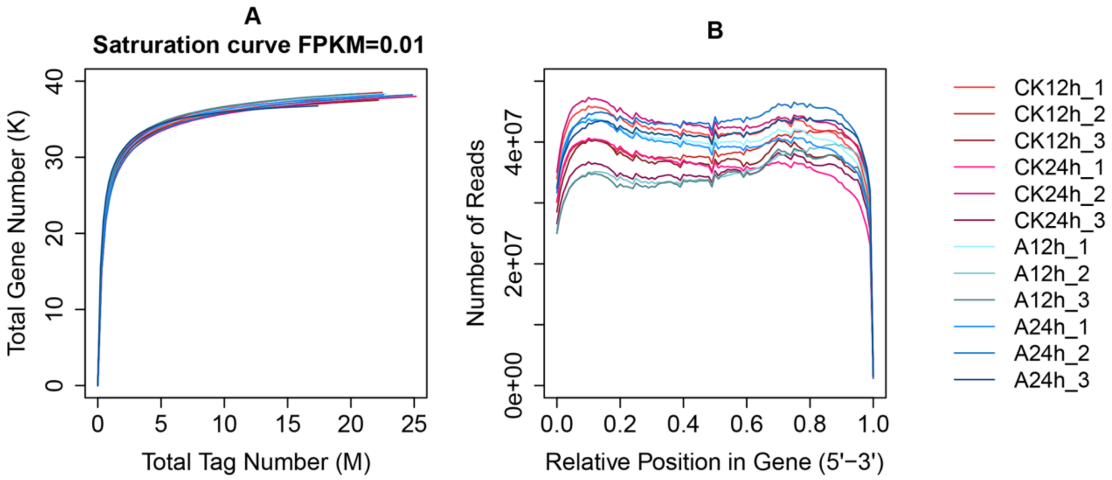

Figure S2 | Sequencing saturation curves (A) and gene coverage analysis (B) of the 12 RNA-Seq samples. Sample names and representative colors are listed on the right.

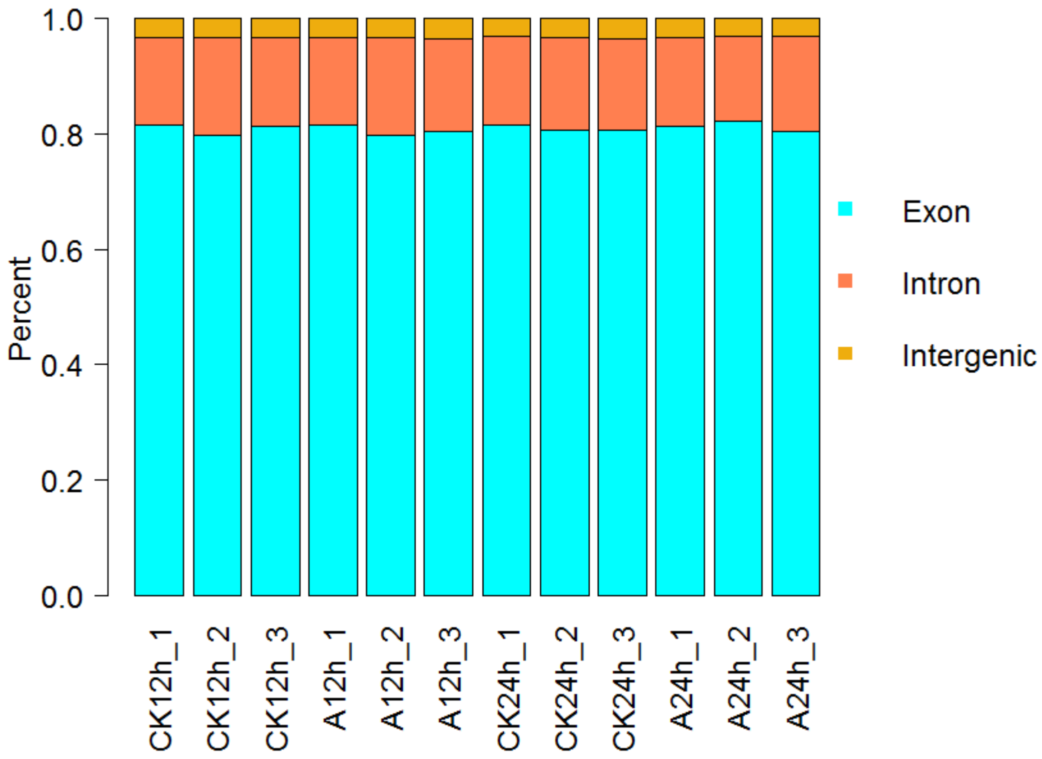

Figure S3 | Distribution of the mapped RNA-seq reads in exon, intron, and intergenic regions.

A: A12h

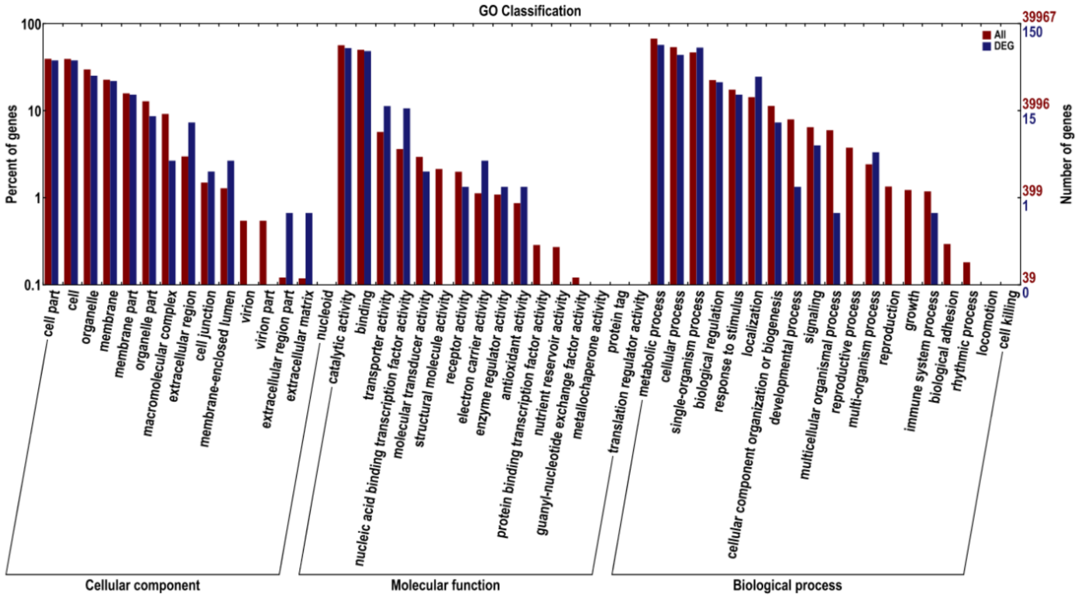

B: A24h

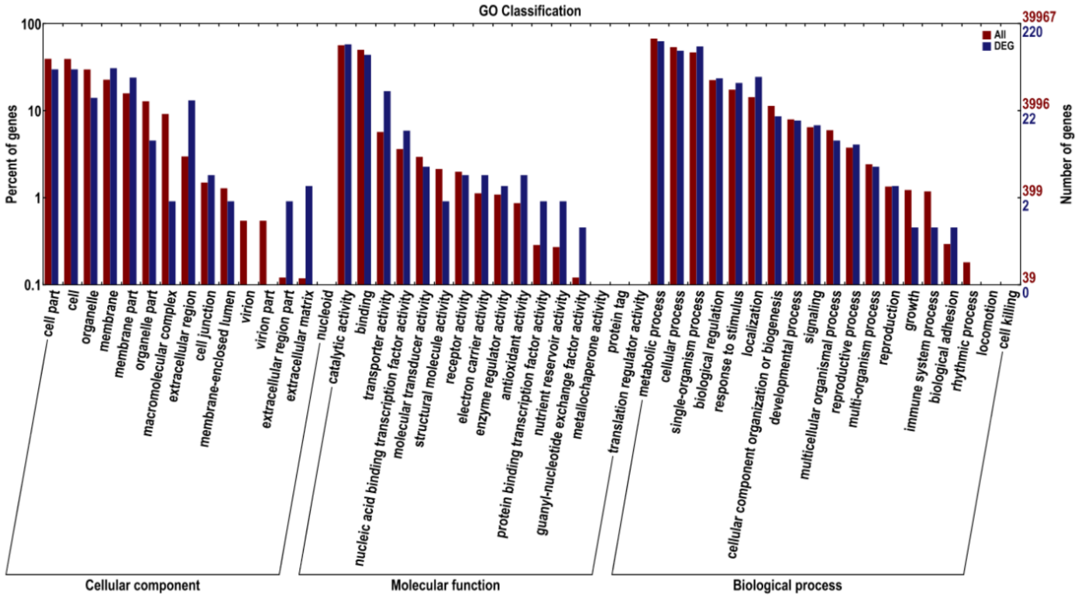

Figure S4 | Functional classifications of DEGs in wild soybean N24852 roots using WEGO. (A) 12 h after 90 mM $NaHCO_3$ (pH = 8.5) treatment. **(B)** 24 h after 90 mM $NaHCO_3$ (pH = 8.5) treatment. The x-axis shows the GO functional categories of cellular components, molecular functions and biological processes. The left y-axis shows the percentage of each category; the right y-axis shows the number of DEGs in each category.

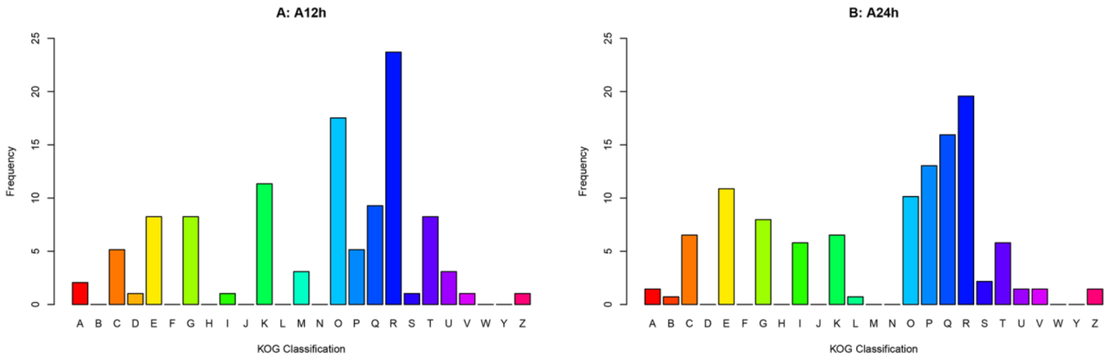

Figure S5 | Functional classifications of DEGs in wild soybean N24852 roots comparing alkaline stress (90 mM $NaHCO_3$, pH = 8.5) with control using KOG at 12 h (A) and 24 h (B). The letters on x-axis represent different classifications. A: RNA processing and modification; B: Chromatin structure and dynamics; C: Energy production and conversion; D: Cell cycle control, cell division, chromosome partitioning; E: Amino acid transport and metabolism; F: Nucleotide transport and metabolism; G: Carbohydrate transport and metabolism; H: Coenzyme transport and metabolism; I: Lipid transport and metabolism; J: Translation, ribosomal structure and biogenesis; K: Transcription; L: Replication, recombination and repair; M: Cell wall/membrane/envelope biogenesis; N: Cell motility; O: Posttranslational modification, protein turnover, chaperones; P: Inorganic ion transport and metabolism; Q: Secondary metabolites biosynthesis, transport and catabolism; R: General function prediction only; S: Function unknown; T: Signal transduction mechanisms; U: Intracellular trafficking, secretion, and vesicular transport; V: Defense mechanisms; W: Extracellular structures; Y: Nuclear structure; Z: Cytoskeleton.

A

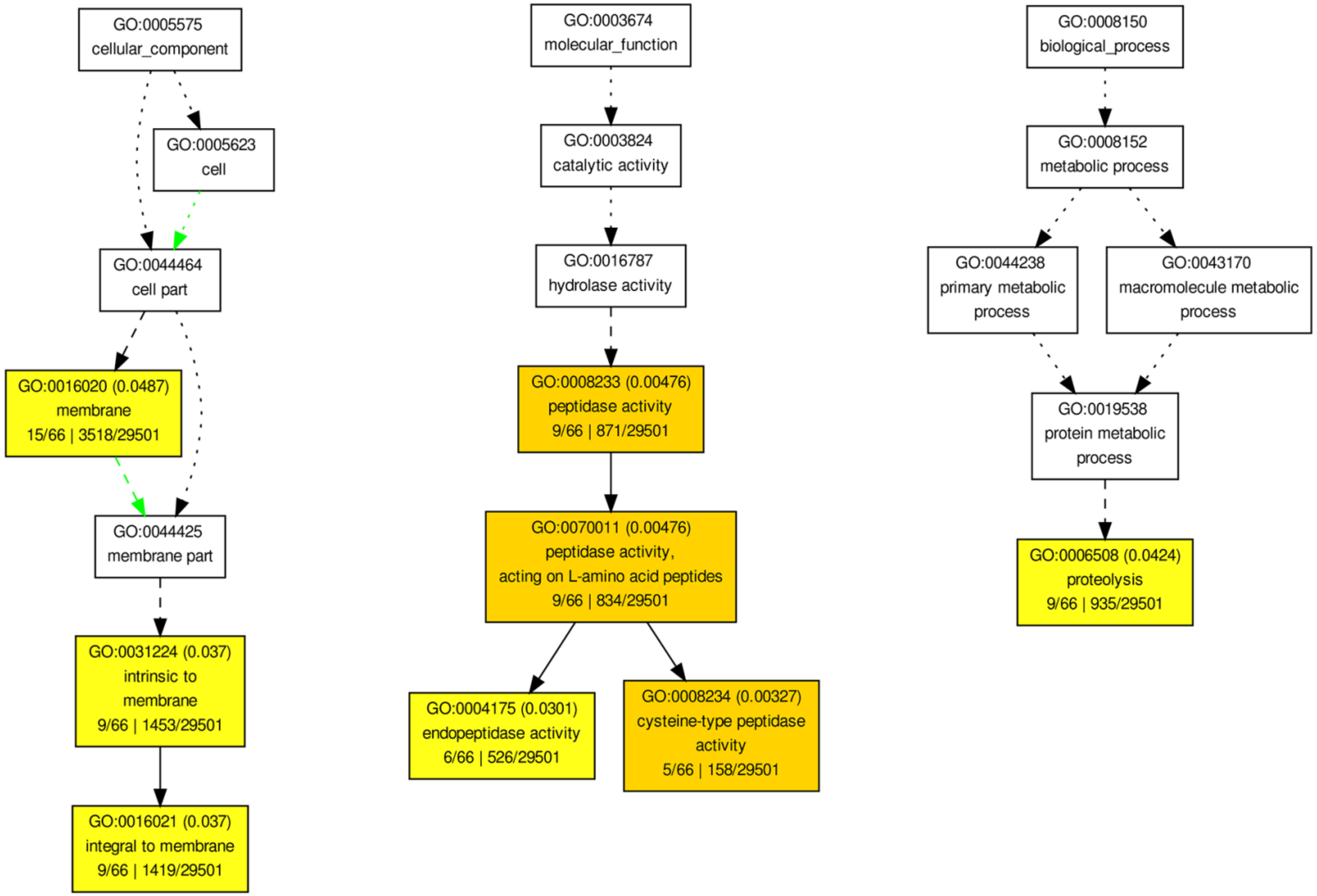
GO:0005575
cellular_component
GO:0005623
cell
GO:0044464
cell part
GO:0016020 (0.0487)
membrane
15/66 | 3518/29501
GO:0044425
membrane part
GO:0031224 (0.037)
intrinsic to
membrane
9/66 | 1453/29501
GO:0016021 (0.037)
integral to membrane
9/66 | 1419/29501
GO:0003674
molecular_function
GO:0003824
catalytic activity
GO:0016787
hydrolase activity
GO:0008233 (0.00476)
peptidase activity
9/66 | 871/29501
GO:0070011 (0.00476)
peptidase activity,
acting on L-amino acid peptides
9/66 | 834/29501
GO:0004175 (0.0301)
endopeptidase activity
6/66 | 526/29501
GO:0008234 (0.00327)
cysteine-type peptidase
activity
5/66 | 158/29501
GO:0008150
biological_process
GO:0008152
metabolic process
GO:0044238
primary metabolic
process
GO:0043170
macromolecule metabolic
process
GO:0019538
protein metabolic
process
GO:0006508 (0.0424)
proteolysis
9/66 | 935/29501

B

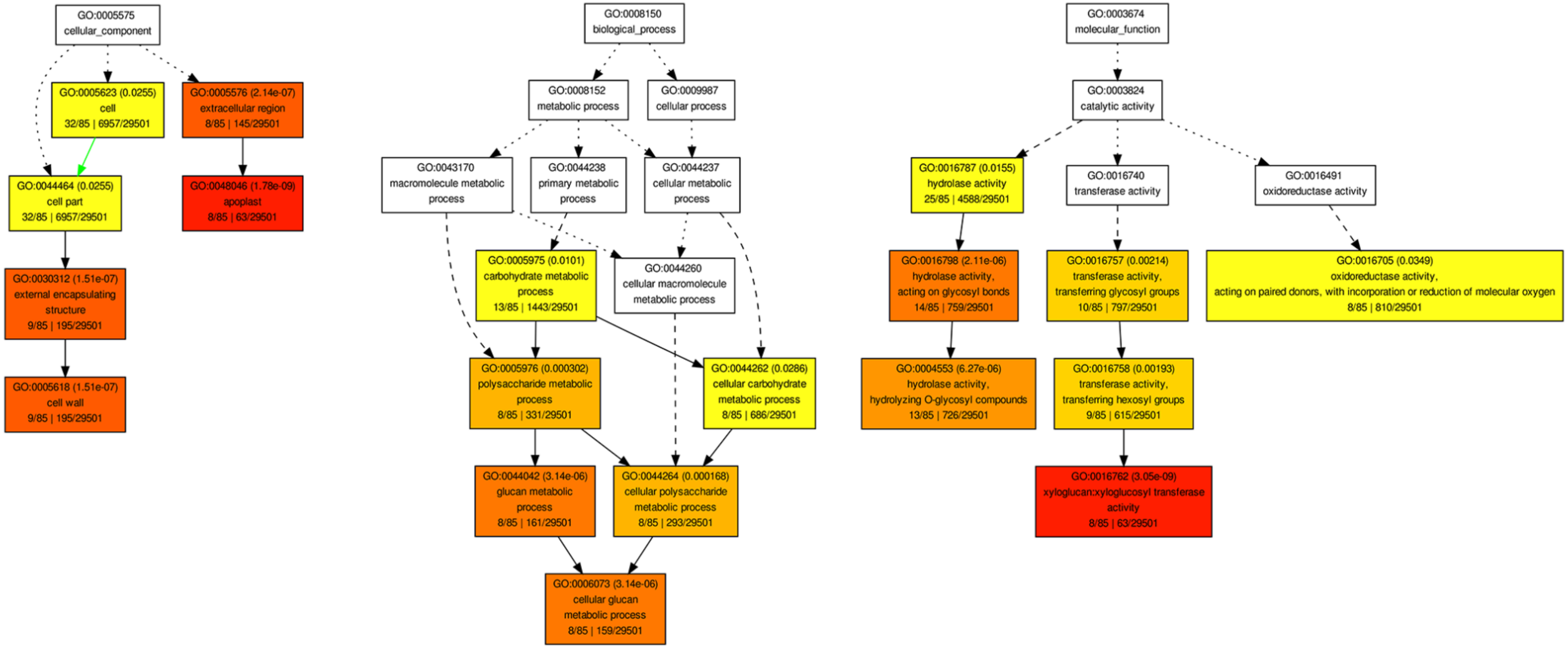

Figure S6 | Enriched Gene ontology (GO) terms for down-regulated genes at 12 h (A) and 24 h (B) after 90 mM $NaHCO_3$ (pH = 8.5) stress. The Singular Enrichment Analysis (SEA) was carried out by agriGO. The GO terms with their ID are written in boxes. The significant (*P* < 0.01, FDR < 0.05) GO terms are in colored boxes (the degree of color saturation is positively correlated to the enrichment level of the GO term), and non-significant terms are in white boxes.

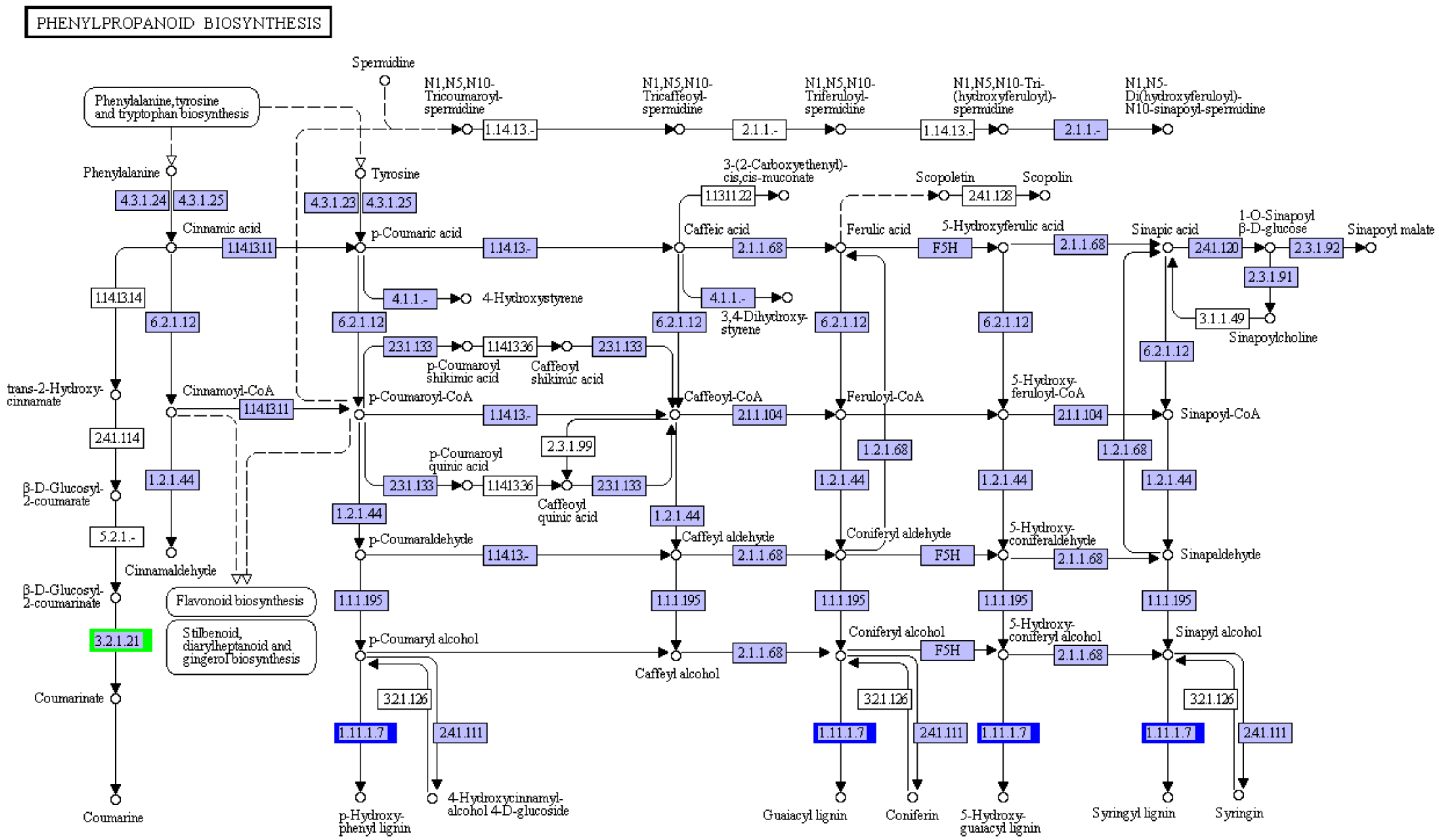

Figure S7 | NaHCO$_3$ stress-responsive genes in phenylpropanoid biosynthesis pathway from KEGG database. Enzymes framed by green, blue indicate that the corresponding genes were down-regulated, both up and down regulated at 24 h after 90 mM NaHCO$_3$ (pH = 8.5) stress, respectively. Down-regulated gene encodes beta-glucosidase (3.2.1.21); Up and down-regulated genes encode peroxidases (1.11.1.7).

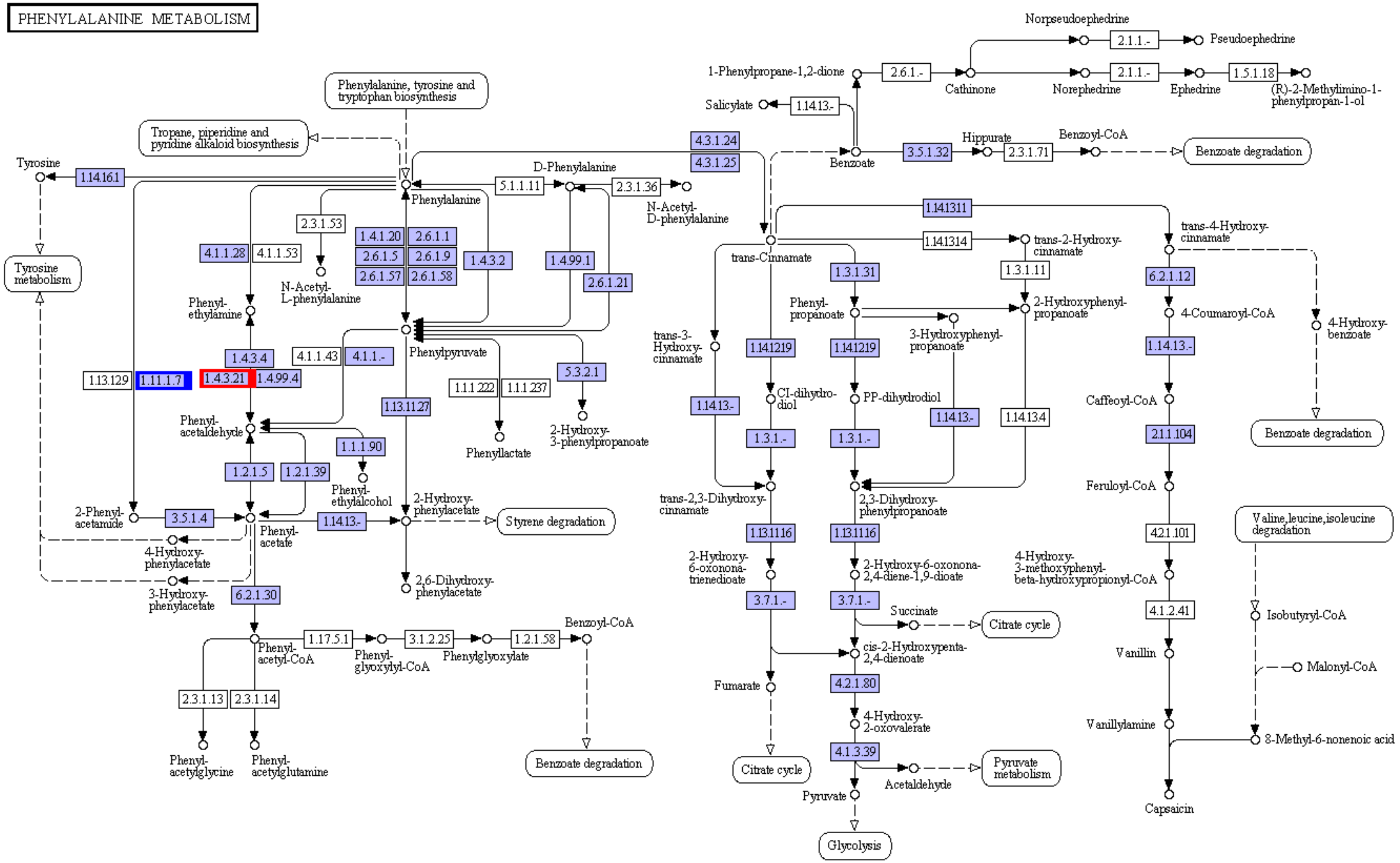

Figure S8 | NaHCO₃ stress-responsive genes in phenylalanine metabolism pathway from the KEGG database. Enzymes framed by red, blue indicate that the corresponding genes were up-regulated, both up and down-regulated at 24 h after 90 mM $NaHCO_3$ (pH = 8.5) stress, respectively. Up-regulated gene encodes primary-amine oxidase (1.4.3.21); Up- and down-regulated genes encode peroxidases (1.11.1.7).
